# Pseudocryptic diversity and species boundaries in the sea cucumber *Stichopus* cf. *horrens* (Echinodermata: Stichopodidae) revealed by mitochondrial and microsatellite markers

**DOI:** 10.1038/s41598-024-54987-w

**Published:** 2024-02-28

**Authors:** Apollo Marco D. Lizano, Kenneth M. Kim, Marie Antonette Juinio-Meñez, Rachel Ravago-Gotanco

**Affiliations:** 1 Faculty of Biosciences & Aquaculture, Nord University, Bodø, Norway; 2 Department of Ecology and Evolution, University of Lausanne, Lausanne, Switzerland; 3 Swiss Institute of Bioinformatics, Lausanne, Switzerland; 4 Marine Science Institute, University of the Philippines, 1101 Diliman Quezon City, Philippines

**Keywords:** Cryptic species, Species boundaries, Sea cucumbers, Stichopodidae, Mitochondrial COI, Microsatellite, Ecology, Evolution

## Abstract

Morphologically cryptic and pseudo-cryptic species pose a challenge to taxonomic identification and assessments of species diversity and distributions. Such is the case for the sea cucumber *Stichopus horrens*, commonly confused with *Stichopus monotuberculatus*. Here, we used mitochondrial cytochrome oxidase subunit I (COI) and microsatellite markers to examine genetic diversity in *Stichopus* cf. *horrens* throughout the Philippine archipelago, to aid species identification and clarify species boundaries. Phylogenetic analysis reveals two recently diverged COI lineages (Clade A and Clade B; *c*. 1.35–2.54 Mya) corresponding to sequence records for specimens identified as *S. monotuberculatus* and *S. horrens,* respectively. Microsatellite markers reveal two significantly differentiated genotype clusters broadly concordant with COI lineages (Cluster 1, Cluster 2). A small proportion of individuals were identified as later-generation hybrids indicating limited contemporary gene flow between genotype clusters, thus confirming species boundaries. Morphological differences in papillae distribution and form are observed for the two species, however tack-like spicules from the dorsal papillae are not a reliable diagnostic character. An additional putative cryptic species was detected within Clade B-Cluster 2 specimens warranting further examination. We propose that these lineages revealed by COI and genotype data be referred to as *Stichopus* cf. *horrens* species complex.

## Introduction

Sea cucumbers (Echinodermata:Holothuroidea) are soft-bodied marine invertebrates found in soft sediment and reef environments from the tropics to temperate regions. Exploited at industrial and artisanal scales, sea cucumbers are a large global invertebrate fishery consisting of multiple species, particularly in the tropics^1^. Increased demand resulted in rapid expansion, serial exploitation, and population declines from overfishing^2,3^. Globally, an estimated 84 species of sea cucumbers are commercially traded^4^.

Species diversity in sea cucumbers is largely underestimated, with species identification confounded by cryptic morphological variation or lack of diagnostic morphological features. ‘Hidden’ diversity, as exemplified by cryptic species or species complexes where two or more distinct species are classified as a single species^5^, or pseudocryptic species where morphological variation has been noted but methodological inadequacies preclude species delineation^6^, represents a challenge to the characterization of biological diversity, and impedes accurate assessments of population- and species-level diversity with critical implications for management and conservation planning^7^. Molecular genetic approaches have contributed greatly to uncovering cryptic species in sea cucumbers. Mitochondrial DNA sequences have revealed cryptic lineages in holothuroids from tropical to polar regions^8–10^, uncovered species complexes in a number of genera, e.g. *Bohadschia*^11,12^, *Holothuria*^13–15^, *Stichopus*^16^, guided taxonomic revisions^17–19^, and enabled the identification of new species^20,21^.

The genus *Stichopus* Brandt, 1835 (Stichopodidae) presently consists of 12 recognized species (WoRMS Editorial Board, 2023)^22^ widely distributed and harvested throughout the Indo-Pacific. Species identification using morphological features can be challenging due to interspecific similarity and intraspecific variability^23^, and is particularly problematic for three species: *Stichopus horrens* Selenka, 1867, *Stichopus monotuberculatus* (Quoy & Gaimard 1834), and *Stichopus naso* Semper 1868, which are commonly misidentified due to their morphological similarities^16,24^. Phylogenetic analysis of mitochondrial cytochrome oxidase (COI) and 16 s rRNA sequences from tropical Pacific specimens provides molecular support for differentiating *S. horrens, S. monotuberculatus,* and *S. naso* as distinct lineages, with *S. horrens* further diagnosed by the presence of tack-like skeletal ossicles (spicules) in the dorsal papillae^16^, consistent with the description of *S. horrens* sensu stricto^23,25^. However, there is considerable uncertainty surrounding the taxonomic identification and associated geographical distribution of *S. monotuberculatus*, with Conand et al.^26^ noting that the species is a western Indian Ocean endemic and Pacific Ocean records are erroneous. Nonetheless, Purcell et al.^4^ refers to *Stichopus* cf. *monotuberculatus* (Quoy and Gaimard 1834) also for samples from the Indo-Pacific, noting that the species described might be different from the original holotype for *S. monotuberculatus* described from the western Indian Ocean, and is probably a complex of subspecies. The lack of DNA sequence data from the *S. monotuberculatus* holotype or from specimens collected from the type locality (Mauritius), has hindered resolution of this taxonomic uncertainty^16^. Considering this, we refer to samples collected in this study as *Stichopus* cf. *horrens,* using the qualifier “cf.” (= confer) to refer to a provisional identification pending confirmation by a specialist of the taxon or comparison with reference material^27^.

Species complexes where at least two cryptic species are identified as a single species, are characterized by unclear species boundaries, and diagnostic phenotypes to distinguish them are either absent or shared among one or more species^7^. While mitochondrial lineages have been used to delineate *Stichopus* species^15,16^, mitochondrial markers alone are inadequate to demonstrate reproductive isolation and define species boundaries^28,29^. Subjecting primary species hypotheses from mitochondrial lineages to further scrutiny using secondary criteria, e.g. additional molecular, biochemical, ecological information^30^ is the underlying principle of integrative taxonomy, with the aim of proposing species hypothesis that are as robust as possible for conversion to formally named taxonomic entities^31,32^. Such rigor is particularly important considering that mitochondrial markers may be particularly limited for species identification in recently diverged lineages such as *S. horrens* and *S. monotuberculatus* (*c*. 0.5–1.0 Million years ago, MYa^16^)*,* where incomplete lineage sorting of ancestral polymorphism, hybridization and introgression may result in mitonuclear discordance, confounding species delimitation^33–36^.

Here we used mitochondrial DNA sequences and multi-locus microsatellite genotypes to test a hypothesis of reproductive isolation among mitochondrial lineages of *Stichopus* cf. *horrens* in the Philippine archipelago. If the mitochondrial lineages represent reproductively isolated groups, we expect: (1) to recover secondary criteria in the form of matching differences in the nuclear genome, with genetically differentiated microsatellite genotype clusters exhibiting minimal admixture consistent with the genotypic species concept^37^; and (2) significant associations between mitochondrial lineage and genotype cluster. We also examine spicule morphology, focusing on tack-like spicules in the dorsal papillae of *S.* cf. *horrens* specimens to evaluate the reliability of this feature as a diagnostic character for species identification.

## Results

### *Stichopus* cf. *horrens* mtDNA lineages and geographic distribution

**Figure 1:**
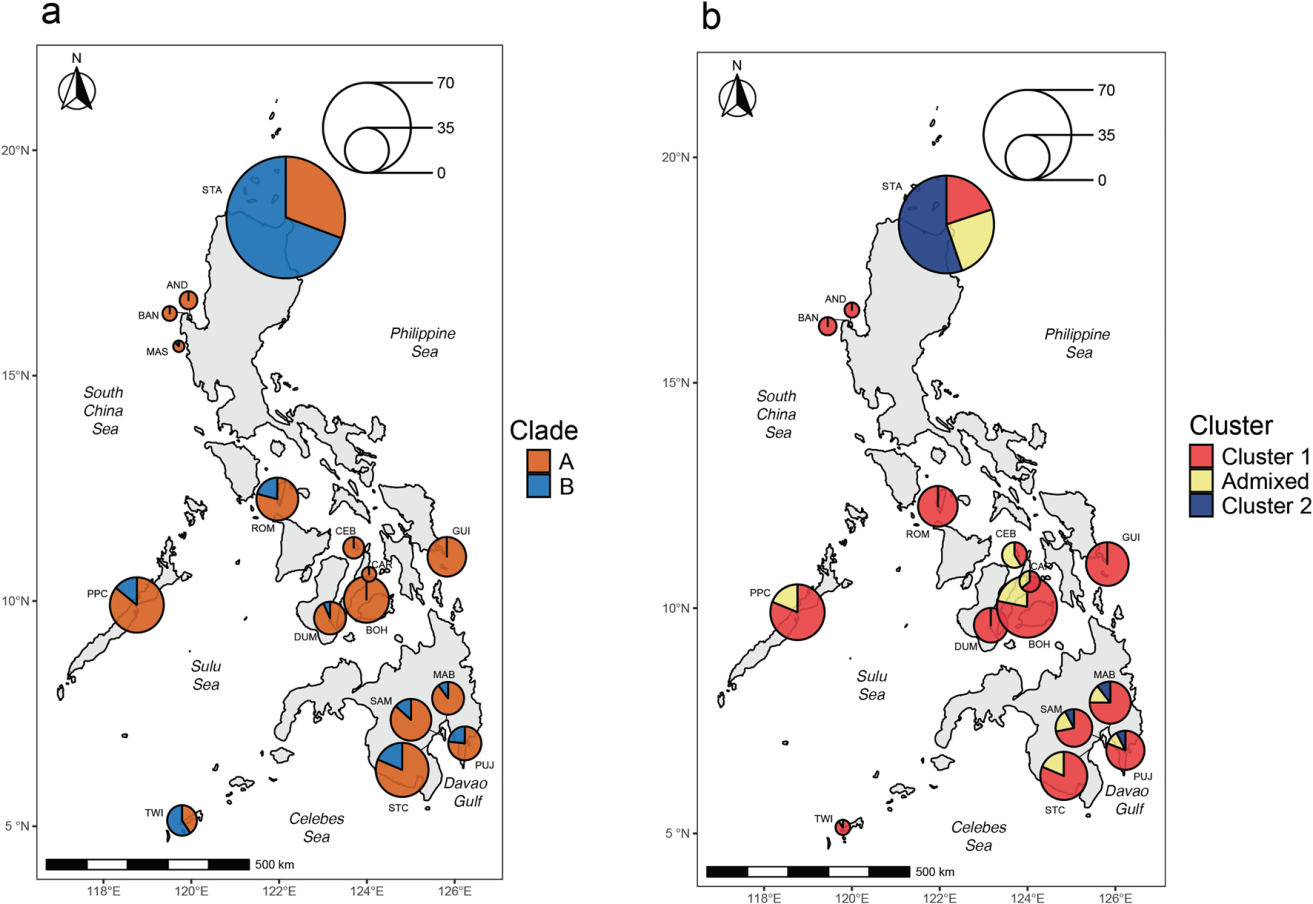
Map showing sampling sites, distribution, and abundance of *Stichopus* cf. *horrens* mitochondrial COI lineages (**a**) and microsatellite genotype clusters (**b**). Pie charts indicate relative abundance of mitochondrial lineages (Clade A and Clade B; 598 samples total) and genotype clusters (Cluster 1, Cluster 2, and Admixed; 396 samples total). Site labels correspond to Table 1. The figures were generated in R using the sf, naturalearth, and ggplot2 packages^105–107^.

*Stichopus* cf. *horrens* were collected from 16 sites across the Philippines (Table 1, Fig. 1). COI sequence data was obtained from 194 individuals. Including COI sequences retrieved from GenBank for *S. horrens* and *S. monotuberculatus* (n = 108 sequences), other *Stichopus* species and Stichopodidae genera *Isostichopus* and *Australostichopus* as outgroups (Supplementary Table S1), the final alignment consisted of 368 sequences with a total length of 502 bp without insertions or deletions, 184 polymorphic sites, collapsed to 98 haplotypes.

**Table 1:** Sample information, genetic diversity indices, mitochondrial COI lineage and genotype cluster assignment for *Stichopus* cf. *horrens.*

Location		Region	Year	Site Code	Lat	Long	mtCOI							Microsatellite			
							N_SEQ_	N_h_	*h*	*Π*	N_RFLP_	Clade A	Clade B	N_G_	C1	C2	A
1	Anda, Pangasinan	South China Sea	2012	AND	16.35	119.94	13	2	0.154	0.000	3	16		10	10		
2	Bani, Pangasinan	South China Sea	2011	BAN	16.25	119.75	11	4	0.691	0.002	2	13		12	12		
3	Masinloc, Zambales	South China Sea		MAS	15.54	119.94	8	7	0.951	0.017		7	1				
4	Sta. Ana, Cagayan	Philippine Sea	2012, 2015	STA	18.51	122.15	38	13	0.851	0.016	70	33	75	65	13	36	16
5	Guiuan, Samar	Philippine Sea	2012	GUI	10.98	125.82	15	2	0.233	0.002	19	34		29	29		
6	Looc, Romblon	Visayan Sea	2013, 2014	ROM	12.26	121.96	21	14	0.938	0.016	17	30	8	27	27		
7	Bantayan, Cebu	Visayan Sea	2013	CEB	11.18	123.70					19	19		17	7		10
8	Carmen, Cebu	Visayan Sea	2014	CAR	10.59	124.05					13	13		14	9		5
9	Tubigon, Bohol	Visayan Sea	2013, 2014	BOH	10.03	123.99					41	41		41	32		9
10	Bais, Dumaguete	Visayan Sea	2012	DUM	9.62	123.16	20	8	0.821	0.007	9	27	2	23	23		
11	Puerto Princesa	Sulu Sea	2013, 2014	PPC	9.91	118.76	19	9	0.801	0.013	30	42	7	39	30		9
12	Tawi-Tawi	Celebes Sea	2012, 2014	TWI	5.13	119.79	25	12	0.910	0.018	2	11	16	10	9		1
13	Samal, Davao	Davao Gulf	2016	SAM	7.00	125.72	8	2	0.389	0.001	28	31	5	25	18	2	5
14	Sta. Cruz	Davao Gulf	2012, 2016	STC	6.85	125.42	16	3	0.575	0.009	32	39	9	32	26		6
15	Mabini	Davao Gulf	2016	MAB	7.30	125.83					29	26	3	28	21	3	4
16	Pujada Bay	Davao Gulf	2016	PUJ	6.84	126.23					30	23	7	27	21	2	3
	Total						194	40	0.861	0.014	344	405	133	396	287	43	66

Sample location includes collection site (Location), Biogeographic Region (Region), year of collection (Year), collection code (Site Code) and georeference (Lat, Long). Number of sequenced individuals (N_SEQ_), number of haplotypes (N_h_), haplotype diversity (*h*); nucleotide diversity (*π*); number of individuals analysed by RFLP for lineage identification (N_RFLP_); number of genotyped individuals (N_G_), and number of individuals assigned to mtCOI lineages (Clade A, Clade B) or genotype clusters (C1 = Cluster 1, C2 = Cluster 2, A = Admixed) are shown.

**Figure 2:**
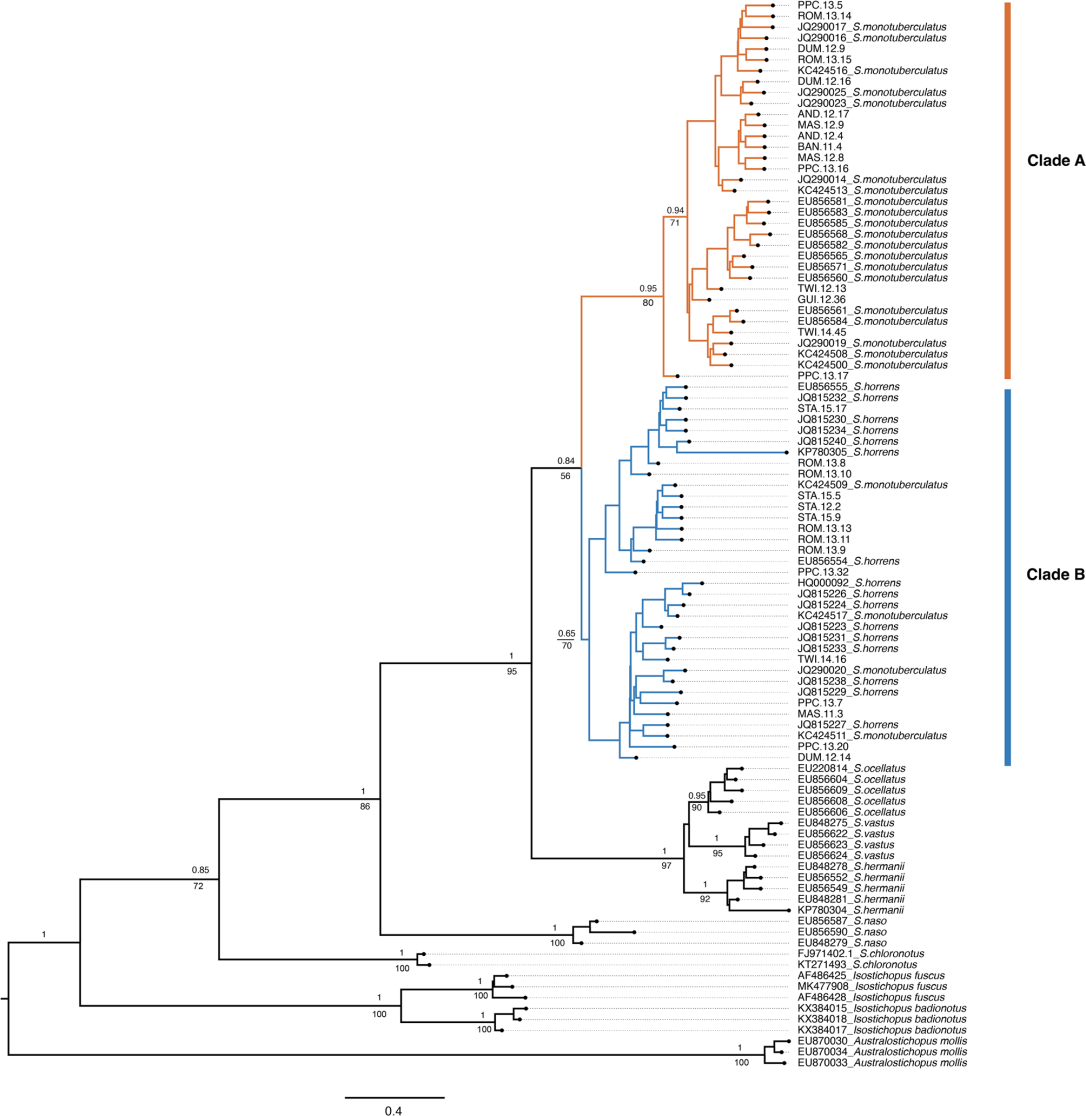
Phylogenetic tree of *Stichopus* obtained by Bayesian inference of mitochondrial COI sequences showing *Stichopus* cf. *horrens* Clade A and Clade B. Outgroup taxa are *Isostichopus* and *Australostichopus*. Posterior probabilities are shown above the branches, and bootstrap values from maximum-likelihood analysis shown below branches.

Maximum Likelihood and Bayesian analyses of the COI dataset grouped *S.* cf. *horrens, S. monotuberculatus,* and *S. horrens* into two well-supported clades hereafter referred to as Clade A and Clade B (Fig. 2). While *S. monotuberculatus* and *S. horrens* were not monophyletic, majority of *S. monotuberculatus* were in Clade A (n = 62 of 71 sequences), while most *S. horrens* were in Clade B (n = 26 of 37 sequences). *Stichopus.* cf. *horrens* Philippine samples were predominantly Clade A haplotypes (n = 138 individuals, 71.1%), while the remainder were Clade B haplotypes (n = 56 individuals, 28.9%). Divergence between the two lineages is estimated at 2.28 MYa (95% HPD 1.34–3.33 MYa) based on the *Isostichopus* calibration node (Supplementary Fig. S1) and 1.35 MYa (95% HPD = 1.18–2.53 MYa) to 2.54 MYa (95% HPD 1.79–3.34 MYa) based on Stichopodidae substitution rates (Supplementary Figs. S2, S3).

**Figure 3:**
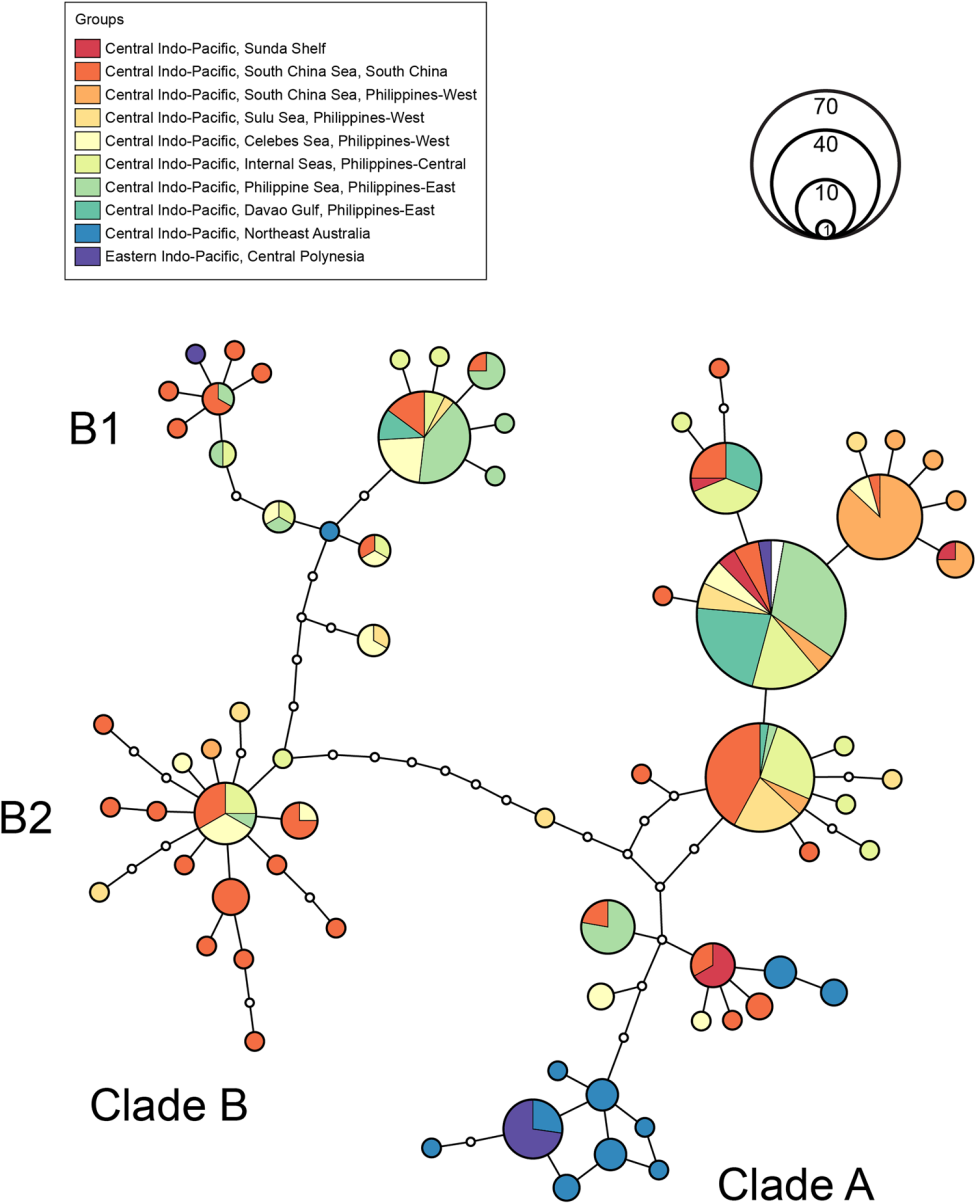
Haplotype network for *Stichopus* cf. *horrens, S. horrens* and *S. monotuberculatus* mitochondrial COI sequences. Each circle represents a haplotype, circle size is proportional to the number of individuals detected. Colors correspond to the sampling locality where each haplotype was detected. White circles connecting sampled haplotypes indicate hypothetical unsampled haplotypes. The network was drawn from 194 *S.* cf. *horrens* (this study), and 108 *S. horrens* and *S. monotuberculatus* CoI sequence records from GenBank with available information on sampling location.

A haplotype network of *S. horrens*, *S. monotuberculatus* and *S.* cf. *horrens* shows Clade A and Clade B haplogroups separated by 7 mutational steps (Fig. 3). Clade A haplotypes exhibit relatively shallow divergence characterized by a few common haplotypes from which singleton or low-frequency haplotypes arise. The Clade B haplogroup meanwhile consists of two subgroups (B1 and B2) separated by 5 mutational steps. Both clades appear to be widely distributed across the Central Indo-Pacific, southwest Pacific and South Pacific Islands, with Clade A relatively more abundant than Clade B (210 and 92 individuals, respectively). Haplotype distribution suggests phylogeographic structure within each clade. For Clade A, a large haplogroup consists of samples from the South China Sea, the Philippine archipelago, and the Sunda shelf, while a small cluster consists of Northeast Australia and Central Polynesia samples. For Clade B haplotypes, Clade B1 consists mostly of eastern region samples (central and eastern Philippines, northeast Australia and central Polynesia) and Clade B2 of mostly western region samples (Indian Ocean, Sunda Shelf, west Philippines) (Supplementary Table S2).

Considering the global sequence dataset of *S. cf. horrens*, *S. horrens,* and *S. monotuberculatus* (n = 302), sequence divergence between Clade A and Clade B is 2.79%. Clade B haplotypes exhibited higher intra-clade divergence (1.16%) than Clade A (0.55%). Clade B haplogroups B1 and B2 are separated by a distance of 1.81%, with relatively low divergence within B1 and B2 groups (0.62% and 0.38%, respectively). Considering only Philippine samples (n = 194), sequence divergence was comparable to the global dataset for Clade A and B (2.67%), Clade B1 and B2 (1.69%), and within Clade A (0.30%), while mean distances were slightly lower within Clade B1 and B2 haplotypes (0.47% and 0.28%, respectively). The frequency distribution of pairwise distances reveals overlapping intra-clade and inter-clade distances, more pronounced for Clade B haplotypes which exhibit a bimodal pattern. Disaggregating Clade B into subclades B1 and B2 reduces the overlap between intra-clade distances (B1, B2) and inter-clade distances (Supplementary Fig. S4).

DNA sequence differences between Clade A and Clade B were observed at 70 sites, 4 of which exhibit fixed nucleotide differences. One of the fixed differences interrupts the recognition sequence for the endonuclease *Rsa*I (COI position 315, nucleotides C/A), which enabled the development of a PCR–RFLP assay for lineage identification based on fragment size differences (Supplementary Fig. S5). Validation of the assay on samples with COI sequence data revealed accurate clade identification for all samples tested (n = 163 individuals). Subsequently, the assay was used to screen additional samples (n = 344), resulting in lineage identification of 538 Philippine samples in total. Clade A haplotypes were more abundant (n = 405 individuals, 75.4% of the sample) and geographically widespread, occurring at all 16 sites while Clade B haplotypes were less abundant (n = 133, 24.6% of the sample), detected at 10 sites (Table 1, Fig. 1).

### Microsatellite genotype clusters and mitonuclear concordance

**Figure 4:**
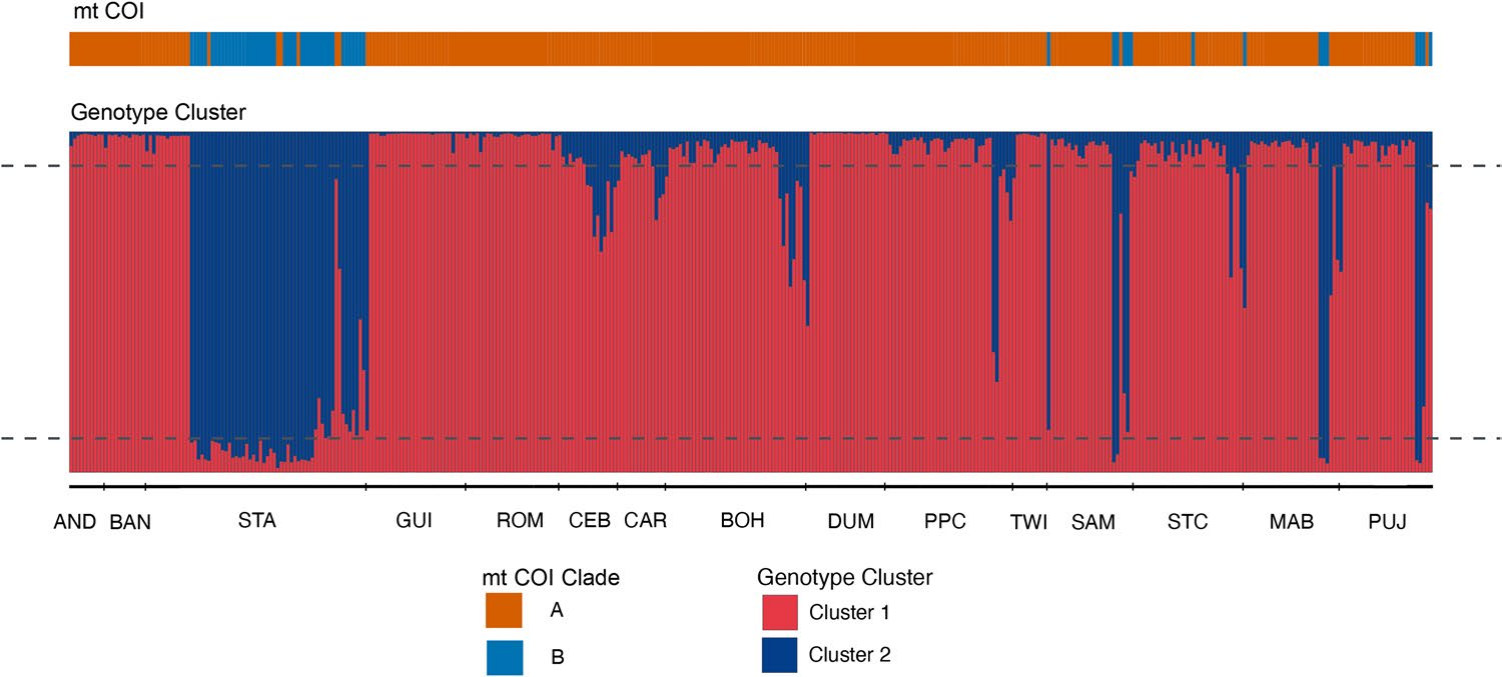
Barplots of mitochondrial COI lineage and microsatellite genotype cluster assignment for *Stichopus* cf. *horrens* individuals (n = 396). Each individual is represented by a vertical bar where the proportion of ancestry is represented by colors corresponding to mitochondrial lineages (Clade A, Clade B) and microsatellite genotype clusters inferred from STRUCTURE analysis when K = 2 (Cluster 1, Cluster 2). The horizontal dashed line represents individual ancestry coefficient (*q*) thresholds for identifying admixed individuals (0.1 < *q* < 0.9).

**Figure 5:**
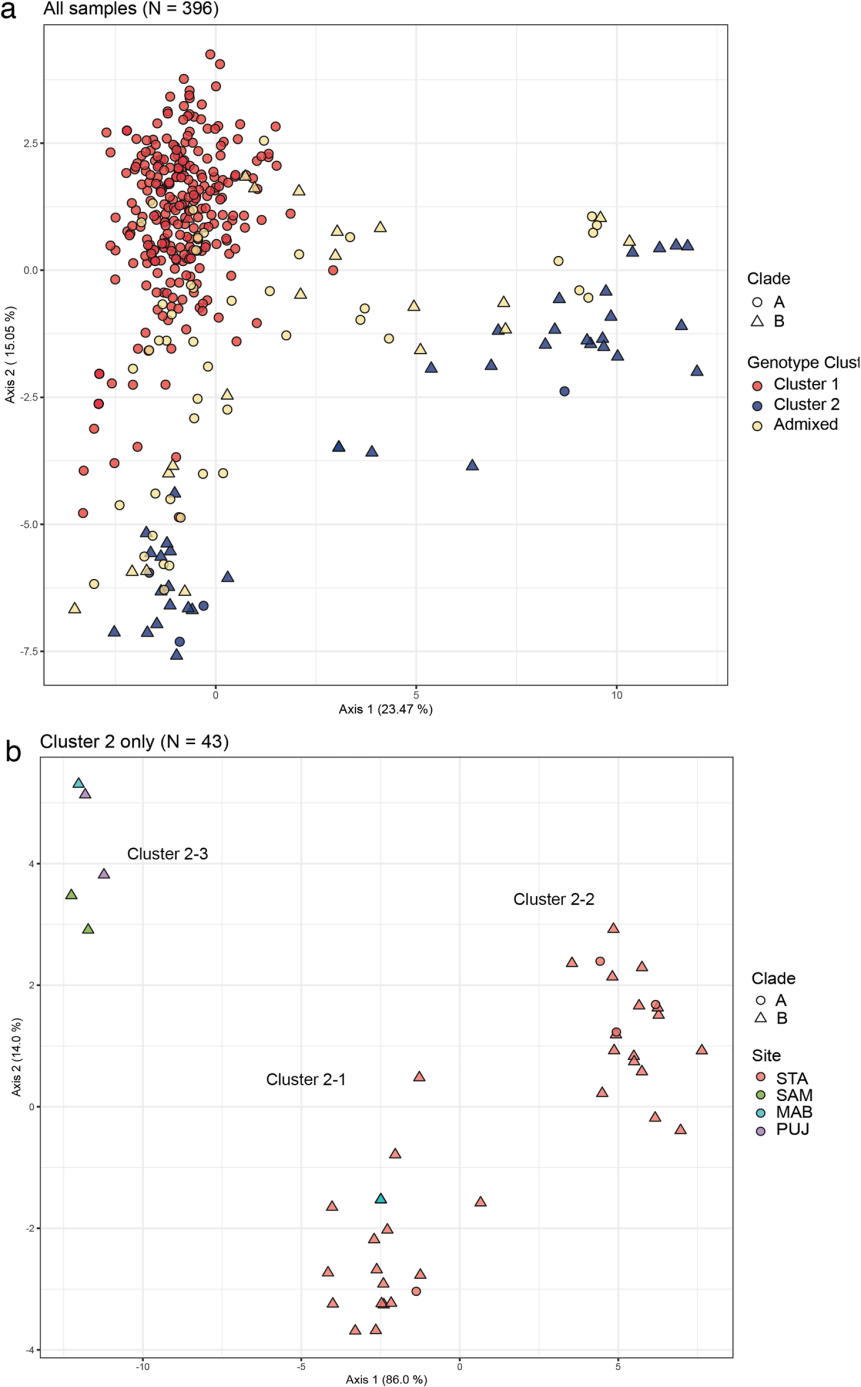
Scatterplot of discriminant analysis of principal components (DAPC) of *Stichopus* cf. *horrens* microsatellite genotypes. DAPC plot of 396 *Stichopus* cf. *horrens* (**a**). Each point represents an individual, with mtDNA lineage indicated by shape and microsatellite genotype cluster based on STRUCTURE analysis indicated by color. DAPC plot of genotype Cluster 2 samples (**b**). MtDNA lineage is indicated by shape and collection site by color.

Genotype data was obtained for 396 *S.* cf. *horrens* individuals at six microsatellite loci. All loci were polymorphic. There was no evidence of large allele dropout for any locus. STRUCTURE analysis revealed the most likely number of clusters to be *K* = 2, hereafter referred to as Cluster 1 and Cluster 2, with 83.3% of the individuals (n = 330) assigned to either of the two clusters based on individual ancestry coefficient threshold of *q* $\ge$ 0.9. The remaining individuals exhibited mixed ancestry (n = 66, 17.7%), and are hereafter referred to as Admixed (Table 2). Cluster assignment is broadly concordant with COI lineages (Table 2, Fig. 4). Majority of Clade A individuals were assigned to Cluster 1 (85.1%, n = 286 of 336 individuals), while Clade B individuals were mostly assigned to Cluster 2 (65%, n = 39 of 60 individuals). A significant association between COI lineage and genotype cluster was detected for Clade A-Cluster 1 and Clade B-Cluster 2 (Fisher’s exact test, *P* < 0.00001), accounting for 82.1% of the total sample. Mitonuclear discordance, i.e. combinations of Clade A-Cluster 2 and Clade B-Cluster 1 was low (1.26%, n = 5). Both clades had individuals exhibiting Admixed genotypes: 13.7% and 14.9% of Clade A and B individuals, respectively. Admixed individuals were observed at 12 of the 15 sampling sites, with four sites accounting for a large proportion of admixed individuals: STA, CEB, BOH, and STC (n = 42 individuals; 63.6% of total admixed individuals). Multivariate analysis using DAPC without a priori grouping of samples revealed broad concordance between COI lineage and genotype cluster. While there is some overlap between Clade A and Clade B individuals, Cluster 1 and Cluster 2 individuals appear as non-overlapping groups, with Admixed individuals occupying an intermediate position between Cluster 1 and Cluster 2 (Fig. 5a).

**Table 2:** Count of mitonuclear combinations for 2 mtCOI lineages and 3 genotype clusters of *Stichopus cf. horrens*.

MtCOI lineage	Genotype cluster			Total
	Cluster 1	Cluster 2	Admixed	
Clade A	286	4	46	336
Clade B	1	39	20	60
Total	287	43	66	396

Comparing STRUCTURE and NewHybrids results show concordant assignments to three genotype classes: two parental classes and putative hybrids (n = 352 of 396 individuals; 88.9%; Supplementary Table S3). Three individuals were not assigned by NewHybrids to any category, while 41 individuals (10.4%) exhibited discordance with STRUCTURE assignment. Discordant assignments consisted of Cluster 1 and Cluster 2 genotypes identified by NewHybrids as F2 hybrids (n = 4 and 22, respectively), and Admixed individuals identified by NewHybrids as parental (n = 15). There were no mis-assignments between parental classes. Considering only individuals with concordant assignments for STRUCTURE and NewHybrids, all Admixed genotypes were identified as F2 hybrids (n = 51). No F1 hybrids were detected.

### Genetic diversity and differentiation

The two mitochondrial lineages exhibit significant genetic differentiation (Φ_*ST*_ = 0.816, *p* < 0.0001). Genetic structure was detected within lineages, with Clade A populations exhibiting greater levels of differentiation than Clade B (Φ_*ST*_ = 0.313, *p* < 0.0001; Φ_*ST*_ = 0.192 *p* < 0.0001, respectively). Haplotype diversity was similar between clades (Clade A, *h* = 0.754; Clade B, *h* = 0.811). Nucleotide diversity was higher for Clade B (π = 0.009) than Clade A (π = 0.003). Population-level haplotype diversity varied widely from *h* = 0.154 (AND) to *h* = 0.938 (ROM).

Genotype clusters likewise exhibit significant differences in allele frequencies over all loci (excluding Admixed individuals: *F*_*ST*_ = 0.099, 95% CI = 0.087—0.114; *F*_*ST*_ corrected for null alleles = 0.095; *G’*_*ST*_, = 0.471), and at each of the 6 loci (Supplementary Table S4). Analysis of statistical power showed that the sample size and six microsatellite loci can detect levels of genetic differentiation as low as *F*_*ST*_ = 0.01 with a high probability (> 95%; Fisher’s exact test, p < 0.0001). Differentiation between genotype clusters is evident even when clusters are sympatric in the Davao Gulf (SAM, MAB, PUJ; overall *F*_*ST*_ = 0.116, range = 0.172–0.270) and in the North Philippine Sea (STA; *F*_*ST*_ = 0.222), with differentiation estimators comparable to allopatric populations (Davao Gulf Cluster 1—STA Cluster 2: *F*_*ST*_ = 0.139, range = 0.137–0.177; Davao Gulf Cluster 2—STA Cluster 1: *F*_*ST*_ = 0.177, range = 0.109–0.216) (Supplementary Fig. S6).

Genotype Cluster 1 and Cluster 2 exhibited significant differentiation among sampling sites, which was more pronounced for Cluster 2 populations (*F*_*ST*_ = 0.159, *G’*_*ST*_, = 0.479; 95% CI > 0) than Cluster 1 (*F*_*ST*_ = 0.067, *G’*_*ST*_, = 0.225; 95% CI > 0). Considering the possibility of further cryptic diversity in Clade B (Clade B1, Clade B2), we performed DAPC on Cluster 2 samples only (n = 43; 4 sites: MAB, PUJ, SAM, STA). Three groups were recovered: two groups of Cayagan samples (STA) designated as Cluster 2–1 and 2–2, and one group of Davao Gulf samples (MAB, PUJ, SAM, with the exception of one individual which occurred in Cluster 2–1), designated as Cluster 2–3 (Fig. 5b). Considering only STA samples, Clusters 2–1 and 2–2 exhibit significant differentiation (*F*_*ST*_ = 0.166, *G’*_*ST*_, = 0.537; 95% CI > 0) at 5 of 6 loci, despite occurring in sympatry. These results suggest additional genotype clusters and cryptic species in STA.

Genetic diversity indices for the genotype clusters show a wide range of allelic diversity across loci ranging from 8 to 34 alleles per locus (Supplementary Table S5). Expectedly, the Admixed cluster exhibited greater allelic richness and heterozygosity than either Cluster 1 or Cluster 2 (*A*_*R*_ = 17.01, 14.24, and 14.36; *H*_*e*_ = 0.851, 0.769 and 0.777, respectively), however the difference was not significant (*A*_*R*_: ANOVA F = 0.456, *P* = 0.642; *H*_*e*_ ANOVA F = 0.544, *P* = 0.591). All loci exhibited private alleles for each of the three clusters.

Identical multilocus genotypes were detected among 396 individuals. Of 377 multilocus genotypes (MLGs) detected, 7 MLGs were observed in more than one individual. The probability that individuals with identical genotypes are the product of sexual reproduction is very low (all *p*_*sex*_ values < 3.1 × 10^–5^, well below the suggested threshold of *p*_*sex*_ = 0.01). Thus, individuals with identical genotypes can be considered as putative clones. Majority of clonal individuals occur in Cluster 1, accounting for six of the seven shared MLGs and 24 of the 26 clonal individuals. Identical genotypes were only observed from the same location, with 3 sites harboring clones: STA (2 MLGs, 5 individuals), GUI (2 MLGs, 15 individuals), and MAB (3 MLGs, 6 individuals).

### Gross morphology and spicule analysis

**Figure 6:**
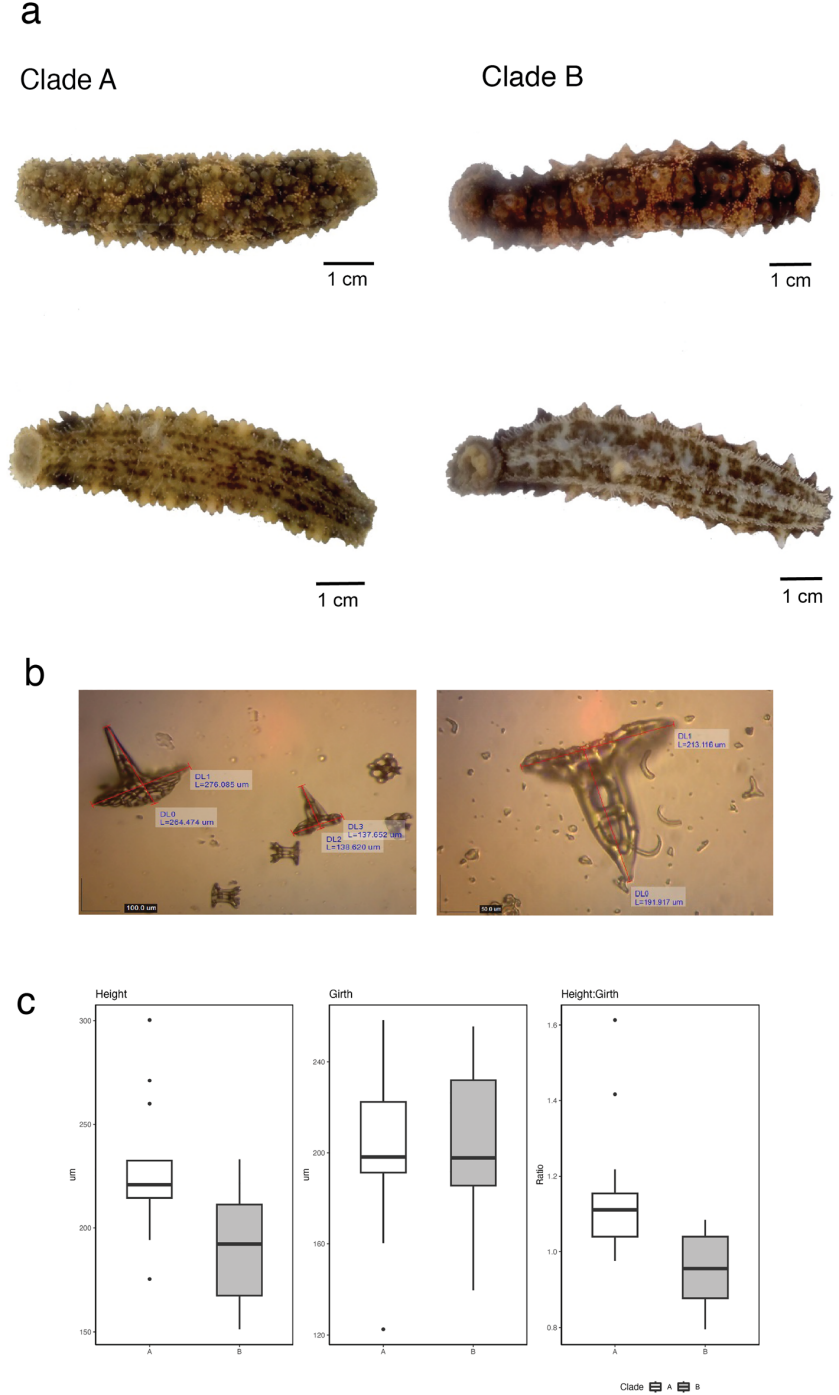
Morphological features of *Stichopus* cf. *horrens*. Photographs of *Stichopus* cf. *horrens* Clade A and Clade B specimens (**a**). Specimens were collected from the same site in Bongao, Tawi-Tawi: TWI.14.50 (Clade A), TWI.14.19 (Clade B). Mitochondrial lineages were identified based on COI sequence data. Image credit: A. Lizano. Images of tack-like spicules from Clade A (left) and Clade B (right) specimens (**b**). Boxplots of tack-like spicule dimensions (height, girth, height:girth) measured from 108 Clade A and Clade B specimens (**c**).

*Stichopus* cf. *horrens* individuals from the two mitochondrial lineages exhibit similarly mottled coloration. The most distinctive differences observed thus far are the shape and distribution of dorsal papillae. Clade A papillae are more densely distributed with a more rounded shape. Clade B papillae are less densely distributed, but are more cone-like, high-spired with a more pointed tip and prominent concentric lines (Fig. 6a).

Dorsal papillae spicules were examined for 108 individuals from 5 sites where Clade A and Clade B are sympatric: Davao Gulf (SAM, STC, MAB and PUJ) and northern Philippine Sea (STA). Tack-like spicules (Fig. 6b) were found in both clades (Clade A = 33, Clade B = 26), and all genotype clusters (Cluster 1 = 31, Cluster 2 = 15, Admixed = 13). The proportion of individuals with tack-like spicules was greater for Clade B (74.2%, 26 of 35 individuals) than Clade A (45.2%, 33 of 73 individuals). Tack-like spicule dimensions, measured from a small sample of 5 individuals per clade (n = 22 tack-like spicules), reveals significant differences. While mean girth was similar between clades (Clade A = 201.12 μm; Clade B = 202.18 μm; t-test *p* = 0.949), Clade A tack-like spicules were significantly longer than Clade B (227.28 μm and 190.91 μm, respectively; t-test *p* = 0.020). The height:girth ratio was likewise different between clades (Clade A = 1.148 μm, Clade B = 0.954 μm; t-test *p* = 0.004) (Fig. 6c).

## Discussion

In this study we report three cryptic species among *Stichopus* cf. *horrens* collected throughout the Philippine archipelago based on evidence from mitochondrial sequences and microsatellite genotypes. *Stichopus* cf. *horrens* mitochondrial lineages Clade A and Clade B (B1, B2) correspond to lineages identified by Byrne and coworkers^16^ as *Stichopus monotuberculatus* and *Stichopus horrens,* respectively. Non-monophyly of GenBank accessions for *S. monotuberculatus* and *S. horrens*, with *S. horrens* occurring in the *S. monotuberculatus* lineage (11 of 37 records), and *S. monotuberculatus* occuring in the *Stichopus horrens* lineage (9 of 62 records) is likely due to imperfect taxonomy or erroneous taxonomic identification, highlighting the challenges of diagnosing these taxa.

Employing sequence divergence thresholds alone poses challenges for uncovering cryptic species within *S.* cf. *horrens*. Sequence divergence among Philippine samples of *S.* cf. *horrens* are comparable with estimates for *S. horrens* and *S. monotuberculatus* from Indonesia^38^ and across the Indo-Pacific^16^. Divergence between Clade A and Clade B (2.67%) is comparable to *S. horrens* and *S. monotuberculatus* (2.70–2.97%), and within-lineage divergence values are likewise comparable for *S. cf. horrens* Clade A (0.30%) and *S. monotuberculatus* (0.30–0.60%), *S. cf. horrens* Clade B (1.69%) and *S. horrens* (1.20%). However, the ratio of inter-clade to intra-clade divergence in *S.* cf. *horrens* (Clade A, B1, B2) falls below the ‘10 × rule’ for delineating putative species^39^, moreover intra-clade distance slightly overlaps with inter-clade distance. This lack of a ‘barcoding gap’ exemplifies the limitations of applying sequence distance thresholds based on single locus markers for designating primary species hypotheses (PSHs) in *S.* cf. *horrens*.

The efficacy of using sequence divergence for species identification is vulnerable to errors due to (1) incomplete taxon sampling which may underestimate intra- and inter-specific variation, and (2) incomplete lineage sorting confounded by introgression among recently diverged or incipient species which could result in overlaps between intra- and inter-lineage divergence^40^. Additional cryptic species within *S.* cf. *horrens* Clade B, consistent with earlier reports of cryptic diversity within the *S. horrens* clade^16^ can account for the low ratio of inter-clade to intra-clade variation. Lumping of cryptic species, exhibited by a multimodal pattern of pairwise genetic distance among Clade B haplotypes, is expected to inflate intra-specific variation and lead to overlap between intra- and inter-clade genetic distances^40^. Recent lineage divergence between Clade A and Clade B, and between subclade B1 and B2 (from late Pliocene to Pleistocene)^41^ coupled with spatial distribution reflecting predominantly eastern and western region haplogroups (Clade B1 and B2, respectively; Fig. 2) may reflect origins from allopatric speciation in the Indo-Pacific region during the Pleistocene. Further studies with expanded geographical sampling are necessary for a more rigorous examination of cryptic diversity within Clade B, and diagnose the possibility of incomplete mitochondrial lineage sorting between Clade A and Clade B.

The recovery of two microsatellite genotype clusters broadly concordant with mitochondrial lineages (PSHs) and exhibiting restricted gene flow even when they occur in sympatry, can be considered as Secondary Species Hypotheses (SSH) supporting delineation of cryptic species within *S.* cf. *horrens*. Where genotype clusters co-occur in the Davao Gulf (SAM, MAB, PUJ) and northern Philippine Sea (STA), levels of genetic differentiation are comparable to allopatric clusters separated by 1,600 kms over which gene flow is highly unlikely. The recovery of two subclusters within Cluster 2 genotypes in STA (Cluster 2–1 and Cluster 2–2; *F*_*ST*_ = 0.174, 95% CI > 0) suggests additional cryptic species within the Clade B-Cluster 2 group. However, due to the small number of Cluster 2 samples, and the lack of genotype data for Clade B2 individuals, we are unable to test for concordance between mitochondrial lineage and genotype cluster within Clade B-Cluster 2.

Further support for limited contemporary gene flow between Cluster 1 and Cluster 2 is indicated by the absence of F1 hybrids and the relatively low proportion of mitonuclear discordance (1.26%). While methods for identifying putative hybrids (STRUCTURE and NewHybrids) exhibited some discordance (10.4%), these were due to assignment of putative hybrids as parentals (Cluster 1, Cluster 2) and vice versa. This discordance is likely due to the sensitivity of NewHybrids to the proportion of hybrid individuals in the sample compared to STRUCTURE^42^, the rarity of private alleles in the admixed individuals, and the confounding effect of population structure observed for both Cluster 1 and Cluster 2. Nonetheless, given the observed discrepancy in identification of hybrids, these results for hybrid identification should be viewed with caution. The use of additional markers which can provide greater resolving power, whether additional microsatellite loci or single nucleotide polymorphisms (SNPs), is strongly recommended for further studies in identifying putative hybrids.

Genetic differentiation between *S.* cf *horrens* genotype clusters (*F*_*ST*_ = 0.099, 95% CI > 0) is comparable to values based on microsatellite data for other cryptic echinoderm species. These include crinoids *Crenolia* sp. (*F*_*ST*_ = 0.152–0.422)^43^, brittle stars, *Ophioderma* (*F*_*ST*_ = 0.19–0.47)^44^, and color variants of the sea cucumber *Apostichopus japonicus* (*F*_*ST*_ = 0.012–0.253)^45^. Pronounced genetic differentiation of recently diverged sympatric lineages of *S.* cf. *horrens* may be due to prezygotic barriers, similar to mechanisms observed in other echinoderms such as rapid accumulation of gametic incompatibilities^46^ or asynchronous spawning^47,48^. In some echinoderms, reproductive isolation inferred based on microsatellite genotypes has been detected despite low levels of COI divergence (< 2% sequence divergence)^49,50^ and low morphological disparity^51,52^. Further examination of morphological variation between *S.* cf. *horrens* Cluster 1 and Cluster 2 is warranted.

Microsatellite genotype data provides insight into the potential association between clonal reproduction and relative abundance of *S.* cf. *horrens* lineages. *Stichopus.*cf. *horrens* Clade A is more widespread and abundant in the Philippine archipelago, similar to *S. monotuberculatus* being more ubiquitous and abundant than *S. horrens* in eastern Australia and Samoa^16^. *Stichopus monotuberculatus’* abundance has been linked to its ability for fissiparous reproduction^16^, similar to another fissiparous stichopodid, *S. chloronotus,* whose population density is positively correlated with annual fission rates^53^. *Stichopus horrens* is not known to exhibit fission. The recovery of identical microsatellite genotypes for *S. cf. horrens* Clade A provides genetic evidence for clonal reproduction in this group. In particular, GUI with the greatest proportion of clonal individuals (50% of the individuals share identical genotypes, all belonging to Clade A), was also where we observed higher densities of *S. cf. horrens*, with some animals exhibiting morphological evidence of fission (I. L. Casilagan, personal observation). Moreover, individuals in GUI were inactive (stationary, body extended) but in exposed habitats during daytime. This is in contrast to other sites where *S. cf. horrens* are typically inactive but sheltering during daytime, a nocturnal activity pattern observed for the species from juvenile stages^54,55^ to adults^56^.

Tack-like spicules are not diagnostic for the two *S.* cf. *horrens* lineages from Philippine specimens. The ossicle form is found in both *S.* cf. *horrens* Clade A (= *S. monotuberculatus* sensu Byrne et al.) and Clade B (= *S. horrens* sensu Byrne et al.), in contrast to previous reports for *S. horrens* and *S. monotuberculatus* lineages examined elsewhere in the tropical Pacific where tack-like spicules have only been observed in *S. horrens*^16^. In Clade A, 49.5% of the specimens had tack-like spicules (30 of 61 Clade A-Cluster 1 samples), at 4 of the 5 sites where individuals from both clades were broadly sympatric (STA, SAM, STC, MAB). Tack-like spicules were absent from all Clade A samples in PUJ (n = 23). Meanwhile, in Clade B, tack-like spicules were not observed in 40% of the specimens (6 of 21 Clade B-Cluster 2 individuals), at all 4 sites where Clade B-Cluster 2 individuals were sampled. The absence of tack-like spicules in some Clade B specimens may be an artifact of incomplete sampling as spicules were examined from only 1 to 2 dorsal papillae per individual. However, the occurrence of tack-like spicules in both clades indicates that it is not a reliable character for lineage identification. Interestingly, the dimensions of tack-like spicules, specifically the height and height:girth exhibit significant differences between Clade A and Clade B. These may be influenced by ontogeny and environmental factors, and a more comprehensive analysis is needed to validate the reliability of spicule dimensions as a diagnostic character.

The cryptic and pseudocryptic diversity observed within *S.* cf. *horrens* across the Philippines warrants a more comprehensive reassessment of the morphology, genetic variation and taxonomy of *Stichopus horrens* across its distributional range. An integrative taxonomic approach is expected to uncover additional differences to delineate additional PSHs and SSHs in *Stichopus* cf. *horrens*. These additional properties include morphological characteristics, reproductive characteristics (timing of spawning, gamete compatibility and fertilization success), chemotaxonomy, ecology, and geographic distribution. Cryptic species occurring in sympatry are more likely to be ecologically differentiated^57^, and examination of sympatric populations of cryptic species of *S.* cf. *horrens* will be crucial to uncover ecological basis for differentiation. Additional genetic data from biparentally inherited nuclear markers such as genome-wide single nucleotide polymorphisms (SNPs) are expected to provide greater resolution for detection of neutral or adaptive genetic differentiation and enable functional annotation and identification of regions of genomic divergence, which can provide insight into mechanisms driving diversification of taxa in early stages of divergence and speciation such as *Stichopus* cf. *horrens.*

Formal recognition and naming of cryptic species is crucial not only for basic research fields that rely on the use of species as units of analysis such as taxonomy and ecology, but also for applied fields with practical implications. Fisheries management and conservation rely on accurate assessments of biological diversity, spatial distributions, and abundance. Delimitation of management units and estimates of demographic connectivity that are not clouded by variability due to cryptic species is essential^32^. Sea cucumbers, *Stichopus* included, are known to produce a diverse repertoire of bioactive compounds^58^ which vary among taxa^59,60^. The existence of undetected cryptic species or inaccurate taxonomy can mask potentially valuable sources for pharmaceutical and industrial applications^61^. For cryptic species where taxonomic identification or nomenclatural revision may take some time to formalize, an increasing number of studies recognize sequence clusters or molecular operational taxonomic units (MOTUs) instead of nominal species as units for taxonomic diversity^62^. Consequently, assigning cryptic specimens to MOTUs or nominal species using methods that can be routinely used even in modest laboratories is of practical importance for species assessments or monitoring. The PCR–RFLP method described here which can differentiate PSHs (Clade A and Clade B) is a practical approach for MOTU identification, at least until more diagnostic morphological features are described to delimit taxa. The potential error in identification using the PCR–RFLP method is estimated to be low based on the discordance between mitochondrial and microsatellite genotype assignments (< 2%). The PCR–RFLP method however has not been developed to differentiate further cryptic genetic variation such as that observed within Clade B or Cluster 2. Currently, variation within Clade B can be interrogated using DNA sequencing, or microsatellite genotyping.

## Conclusions

In this study, pseudocryptic diversity within *Stichopus* cf. *horrens* was clarified using molecular data, with primary species hypotheses delineated by mitochondrial lineages and genotype clusters identified by microsatellite markers used as secondary species hypotheses to delineate species boundaries in the absence of unambiguous distinguishing morphological characters. The broad concordance between highly differentiated genotype clusters and mtDNA lineages, coupled with the absence of F1 hybrids indicates restricted gene flow and limited contemporary interbreeding, providing further support for species delineation. Tack-like spicules are not a reliable character for lineage identification, and more detailed morphological examination is warranted to uncover other potentially diagnostic morphological characters. In addition to providing additional genetic basis to support the delineation of *S.* cf. *horrens* Clade A and Clade B as valid species, the possibility of further cryptic speciation within *S.* cf. *horrens* Clade B is reported, based on a larger sample size of mitochondrial sequences from the Philippines coupled with microsatellite data. Considering the taxonomic uncertainty and confusion regarding the identification of specimens collected from the Pacific region as *Stichopus monotuberculatus* (Quoy & Gaimard 1834), we propose that the broader *S.* cf. *horrens* Clade A and Clade B lineages, and additional putative cryptic species contained therein, be referred to as *Stichopus* cf. *horrens* species complex until a nomenclatural revision is forwarded and unequivocal Linnean names are assigned. Referring to the *S.* cf. *horrens* species complex recognizes cryptic diversity, as well as accommodates species uncertainty without creating further taxonomic confusion.

## Methods

### Sample collection

*Stichopus* cf. *horrens* were collected from representative sites spanning different marine biogeographic regions across the Philippine archipelago (Table 1, Fig. 1). Morphological identification followed species identification guides for sea cucumbers^63,64^, taxonomic descriptions and species keys^23,25^. Dorsal papillae were collected from each individual and preserved in 95% ethanol for genetic analysis. For spicule analysis, one to two dorsal papillae per specimen were incubated in 10% bleach for up to 30 min. Spicules were examined using a digital microscope to search for tack-like spicules reportedly diagnostic for *S. horrens* sensu stricto^23,25^, for representative specimens from *S.* cf. *horrens* mitochondrial lineages. Spicule dimensions were measured using DinoCapture 2.0.

### DNA Extraction, PCR amplification, sequencing and genotyping

Total DNA was extracted using a Chelex-proteinase K method^65^, followed by CTAB extraction^66^. Extracted DNA was examined for quality and concentration using a combination of agarose gel electrophoresis and spectrophotometric methods. A portion of the mitochondrial COI region was amplified using primers COIef and COIer^67^. Reactions consisted of 1X PCR buffer, 2.5 mM MgCl_2_, 0.1 μM of each primer, 0.025 units/uL *Taq* polymerase (Invitrogen), and 1 µL DNA template in a final volume of 25 uL. Amplification was performed under the following conditions: initial denaturation at 94 °C for 5 min, 35 cycles of denaturation at 94 °C for 60 s, annealing at 54 °C for 90 s and extension at 72 °C for 60 s, with a final extension at 72 °C for 5 min. PCR products were purified by incubation with 6 units of Exonuclease I (New England Biolabs) and 0.6 units of Antarctic Phosphatase (New England Biolabs) at 37 °C for 60 min, then 80 °C for 20 min to remove unincorporated primers and nucleotides. Bidirectional nucleotide sequencing was performed (1st Base sequencing, Malaysia and Macrogen, Korea). Sequence proofreading and contig assembly was conducted using Geneious 6.1.6^68^.

Microsatellite markers developed for *S. horrens*^69^ and *S. monotuberculatus*^70^ were tested for amplification. Five loci developed for specimens identified as *S. horrens* were not consistently amplified across samples (*Sh003, Sh006, Sh007, Sh013* and *Sh015*). Thus, only six microsatellite markers developed from specimens identified as *S. monotuberculatus* were used for further genotyping and were amplified in two separate multiplex reactions: multiplex 1 (*Sm001, Sm007, Sm010, Sm012*) and multiplex 2 (*Sm011, Sm013*). Each multiplex reaction consisted of 1 × Qiagen Multiplex PCR Master Mix, 0.2 μM of each primer (5’-fluorophore labelled forward primer and reverse primer), 0.5 μL template DNA, and distilled deionized water to a final volume of 10 μL. Amplification was performed under the following conditions: initial denaturation at 94 °C for 5 min, 35 cycles of denaturation at 94 °C for 60 s, annealing at 57 °C for 90 s and extension at 72 °C for 90 s, with a final extension at 72 °C for 30 min. PCR products from multiplex reactions for each individual were pooled, mixed with fluorescent size standard (GeneScan™ LIZ®-500, Applied Biosystems Inc.), Hi-Di™ Formamide (Applied Biosystems Inc.) and deionized water to a final volume of 10 μL. Fragments were separated on an ABI 3730xl (Cornell University, Life Sciences Core Laboratories Center). Allele calling was performed using the microsatellite plug-in within Geneious v.6.1.6. The dataset was examined for genotyping errors and null alleles using MicroChecker^71^.

### Phylogenetic analysis and divergence time estimation

A COI sequence dataset was assembled which included *Stichopus* cf. *horrens* sequences generated from this study, and homologous sequences for *Stichopus* spp. retrieved from GenBank following a Basic Local Alignment Search Tool (BLAST) query. Sequences from other Stichopodidae genera were included as outgroups: *Isostichopus badionotus* (Selenka, 1867)*, Isostichopus fuscus* (Ludwig, 1875)*,* and *Australostichopus mollis* (Hutton, 1872). Sequences were edited, aligned, and managed using Geneious Prime 2023.1 (https://www.geneious.com).

Phylogenetic relationships among haplotypes were inferred using maximum likelihood (ML), and Bayesian approaches. The optimal nucleotide substitution model was determined using Modeltest-NG v0.1.5^72^ run on the CIPRES portal^73^. The HKY + I + G model was identified as the best-fit model based on Bayesian information criterion (BIC) and the Akaike information criterion (AIC). The ML analysis was performed using RAxML-NG^74^, with support estimation for the best tree calculated from 5000 bootstrap replications. Bayesian inference was performed with MrBayes v3.2.7^75^ run on CIPRES. Two independent runs were performed, each consisting of four Markov chain Monte Carlo (MCMC) chains sampled every 1,000 generations for 10,000,000 generations. Runs were checked for convergence using Tracer v1.7^76^. Tree files from the two runs were combined to reconstruct a maximum clade credibility tree using TreeAnnotator^77^ (https://www.beast2.org/treeannotator/), discarding the first 5000 trees (burn-in = 25% of saved trees). Haplotype networks were calculated using the TCS statistical parsimony algorithm^78^ implemented in TCS v1.2.1^79^. The raw graph output of TCS was visualized using the web implementation of tcsBU^80^ and edited for publication in Adobe Illustrator.

Lineage divergence times were estimated using BEAST v2.6.3^81^ run on CIPRES. Since Stichopodidae have a poor fossil record^82^, we used a molecular clock calibration using substitution rate estimates for the COI region specific for Stichopodidae (1.81% per million years; Byrne et al.^16^), and the maximum rate inferred for echinoderm mitochondrial protein coding regions (3.39% per million years^83^). For each substitution rate, three independent runs of 50 × 10^–6^ MCMC generations each were performed using an HKY + G + I substitution model with 4 gamma categories, G = 1.25, I = 0.51, a strict molecular clock, and a Yule tree prior. Divergence time was also estimated using the *I. badionotus-I. fuscus* node as a calibration point on the assumption that these two taxa are geminate species following the closure of the Isthmus of Panama at 3.1 MYa^84^. The same parameters were used for the substitution model, clock models (strict clock), and run length, with the *Isostichopus* node set as monophyletic, following a log normal distribution with mean of 0.1 and offset of 3.1 MY. For each run, convergence was assessed by checking logged statistics using Tracer. Tree files were combined using LogCombiner v2.3.1^81^, and the maximum clade credibility tree with mean divergence times was reconstructed using TreeAnnotator v1.7^77^ with a burn-in of 20%. Trees were visualized using FigTree v1.4^85^.

### Inferring microsatellite genotype clusters

To test a hypothesis of reproductive isolation between COI lineages of *S.* cf. *horrens*, genetic groupings based on microsatellite data were examined using two approaches: multivariate analysis and model-based assignment. Multivariate analysis was performed using a discriminant analysis of principal components (DAPC) implemented in the R package ‘adegenet’ v2.1.3^86^. This analysis transforms the data using a principal component analysis (PCA) followed by discriminant analysis (DA) producing synthetic discriminant functions that maximize between-group variation while minimizing within-group variation^87^. To avoid issues of overfitting, the number of principal components (PCs) to retain was chosen following a cross-validation analysis implemented using the *xvalDAPC* function using 500 replicates. The number of genetic clusters (*K*) in the dataset was assessed using the *find.clusters* function which implements a sequencing *k-*means clustering algorithm, with the best-supported number of clusters determined based on the BIC value. A Bayesian model-based assignment method implemented in STRUCTURE v.2.3.4^88^ was also used to infer the number of genetically homogeneous clusters (*K*) which minimize departures from Hardy–Weinberg equilibrium and linkage disequilibrium and calculate the probability of individual membership in each cluster *K* (individual ancestry coefficients, *q*). Ten replicate MCMC simulations were performed for each value of *K* (*K* = 1 to 15), using a model with admixture and correlated allele frequencies^89^. Each run was carried out for 1 × 10^6^ iterations with an initial burn-in of 100,000 steps. The most likely number of clusters was inferred based on the Δ*K* method^90^ implemented in Structure Harvester v0.6.94^91^, available online (http://taylor0.biology.ucla.edu/structureHarvester/). Ancestry coefficients for each value of *K* were averaged across replicate runs using CLUMPP v1.1.2^92^. To assign an individual to a cluster, we used a threshold value of *q* ≥ 0.9 following Vaha and Primmer^42^, while individuals having a *q* value between 0.10 and 0.9 were categorized as admixed.

To complement STRUCTURE results in identifying putative hybrids or admixed individuals, additional analysis was performed using NewHybrids v.1.1^93^. NewHybrids assumes that the sample is drawn from a mixture of parental and admixed individuals, and estimates the posterior probability, *q*_*i*_, that an individual belongs to one of six genealogical classes: two parental classes (P1, P2), and 4 hybrid categories (F1, F2, F1 × P1 and F1 × P2). This is consistent with a scenario where mitochondrial lineages represent two reproductively isolated groups, and matching differences are reflected in the microsatellite genotypes (*K* = 2). NewHybrids was run using the following parameters: a burn-in period of 100,000 steps and 1 × 10^6^ MCMC steps, no prior population information, and Jeffrey-type priors. Individuals were assigned to either Parental 1 (P1), Parental 2 (P2), first generation hybrid progeny (F1), second generation hybrid progeny (F2), and backcrosses (F2 × P1, F2 × P2), using a threshold of *q*_*i*_ > 0.5. To assess the accuracy of NewHybrids in identifying hybrid individuals, hybrid genotypes were simulated using HYBRIDLAB 1.0^94^. Parental genotypes (P1, P2) were selected based on STRUCTURE analysis (threshold *q* ≥ 0.9) and used in the simulations. A total of 1200 individuals were simulated (200 individuals per genealogical class) and analyzed.

### Genetic diversity and genetic differentiation

Genetic diversity estimates were calculated for mtDNA sequences and microsatellite data. For COI sequences, the number of haplotypes (N_h_), haplotype diversity (*h*), nucleotide diversity per site (π), and number of polymorphic sites (P) for each population were calculated in Arlequin v3.5.2^95^. For microsatellite genotypes, R^96^ packages were used in RStudio to perform exact tests of departures from Hardy–Weinberg equilibrium expectations for locus-population combinations (‘genepop’ v1.1.7^97^), genetic diversity estimators such as the number of alleles, allelic richness, observed and expected heterozygosity (*H*_*O*_, *H*_*E*_) and the inbreeding coefficient (*F*_*IS*_) (‘diveRsity’ v1.9.9^98^), and genotypic diversity estimated by the number of multilocus genotypes (‘poppr’ v2.9.3^99^). Significance levels for multiple HWE tests were adjusted following a correction for false discovery rate^100^.

Genetic differentiation was measured using estimators of Wright’s fixation index (*F*_*ST*_)^101^. For sequence data, we used Φ_*ST*_ which incorporates sequence divergence among haplotypes, calculated using Arlequin. For multilocus genotype data, Weir and Cockerham’s *F*_*ST*_^102^, as well as a standardized *G*_*ST*_ value (Hedrick’s *G’*_*ST*_^103^) which accounts for heterozygosity of highly polymorphic microsatellite loci, were calculated using ‘diveRsity’ v1.9.9. The significance of *F*_*ST*_ and *G’*_*ST*_ (null hypothesis of genetic homogeneity, H_o_: *F*_*ST*_ = 0) was evaluated by estimating the bootstrapped 95% confidence interval (95% CI). Statistical power to detect genetic heterogeneity at various levels of true differentiation (*F*_*ST*_) among clusters was estimated using the program POWSIM^104^.

### Supplementary Information

Supplementary Figures.

Supplementary Information 2.

Supplementary Information 3.

## Data Availability

COI sequence data are deposited in GenBank under nucleotide Popset 1,189,490,435 and Accession Numbers OR468389–OR468546. Microsatellite genotype data and associated metadata are deposited in Zenodo (10.5281/zenodo.8273196).
