# Supplementary material for: Pseudocryptic diversity and species boundaries in the sea cucumber Stichopus cf. horrens (Echinodermata: Stichopodidae) revealed by mitochondrial and microsatellite markers
Source: Sci Rep. 2024 Feb 28;14:4886. doi: 10.1038/s41598-024-54987-w (PMC10901784; doi:10.1038/s41598-024-54987-w)
Supplement: Supplementary file 1 — Supplementary Figures. [file 41598_2024_54987_MOESM1_ESM.pdf]

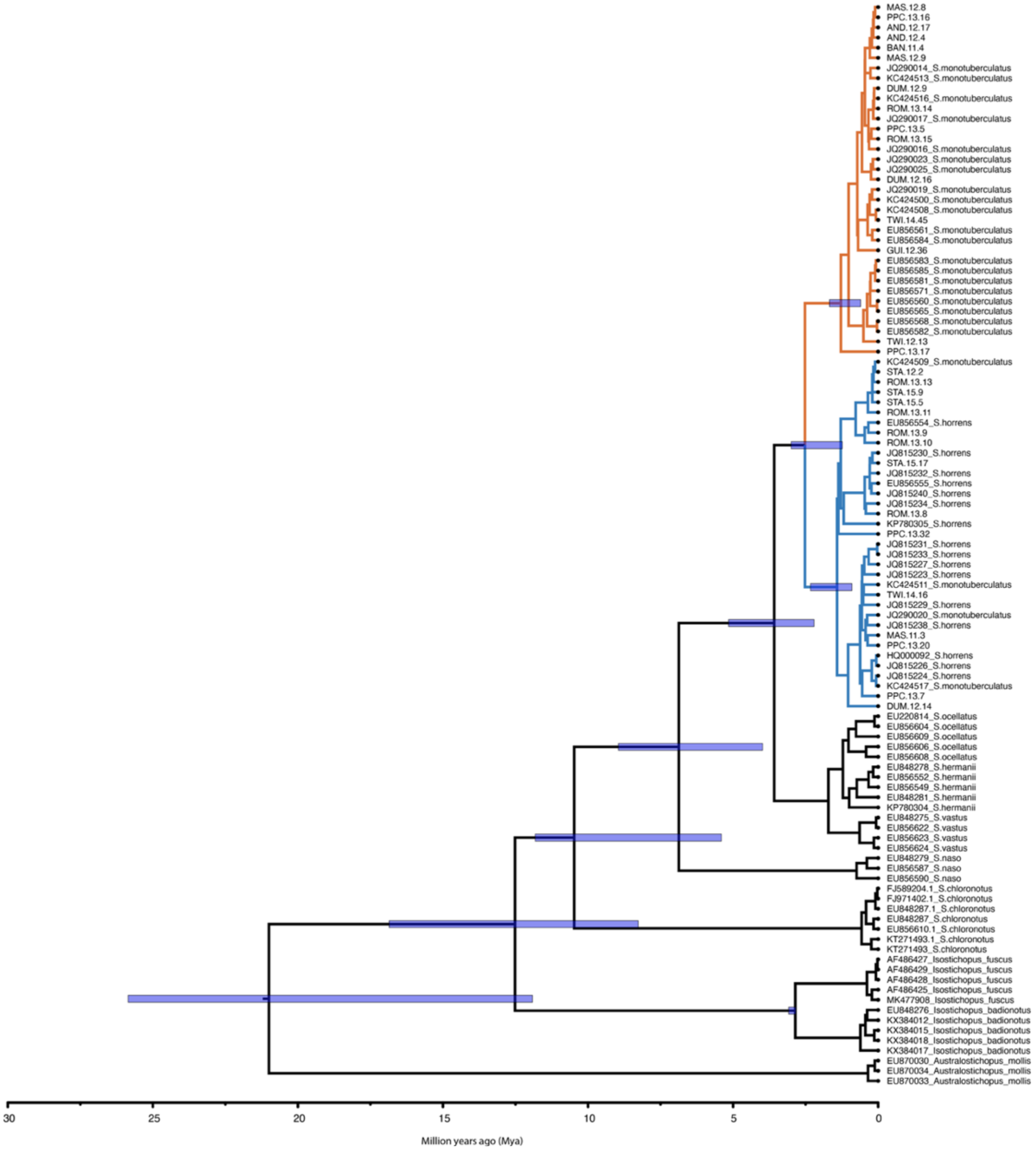

Supplementary Figure S1. Chronogram of *Stichopus* cf. *horrens, S. horrens,* and *S. monotuberculatus* resulting from analysis of the mitochondrial cytochrome oxidase I region in BEAST, displaying mean divergence time and 95% confidence interval (blue bar). Timescale is calibrated based on *Isostichopus badionotus* and *Isostichopus fuscus* divergence during the closure of the Isthmus of Panama (3.1 Million years ago).

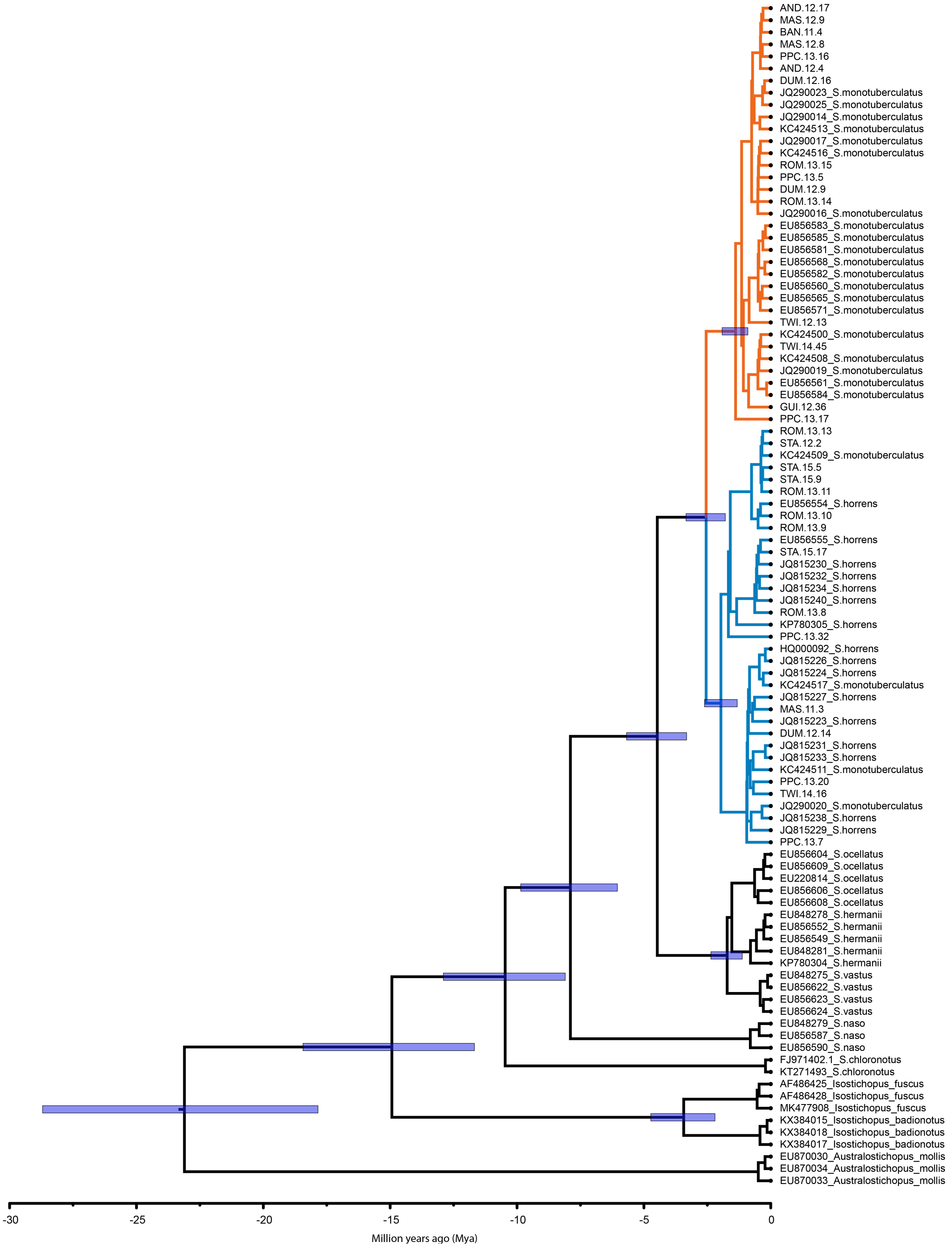

Supplementary Figure S2. Chronogram of *Stichopus* cf. *horrens, S. horrens,* and *S. monotuberculatus* resulting from analysis of the mitochondrial cytochrome oxidase I region in BEAST, displaying mean divergence time and 95% confidence interval (blue bar). Timescale is based on a substitution rate of 1.8% per million years.

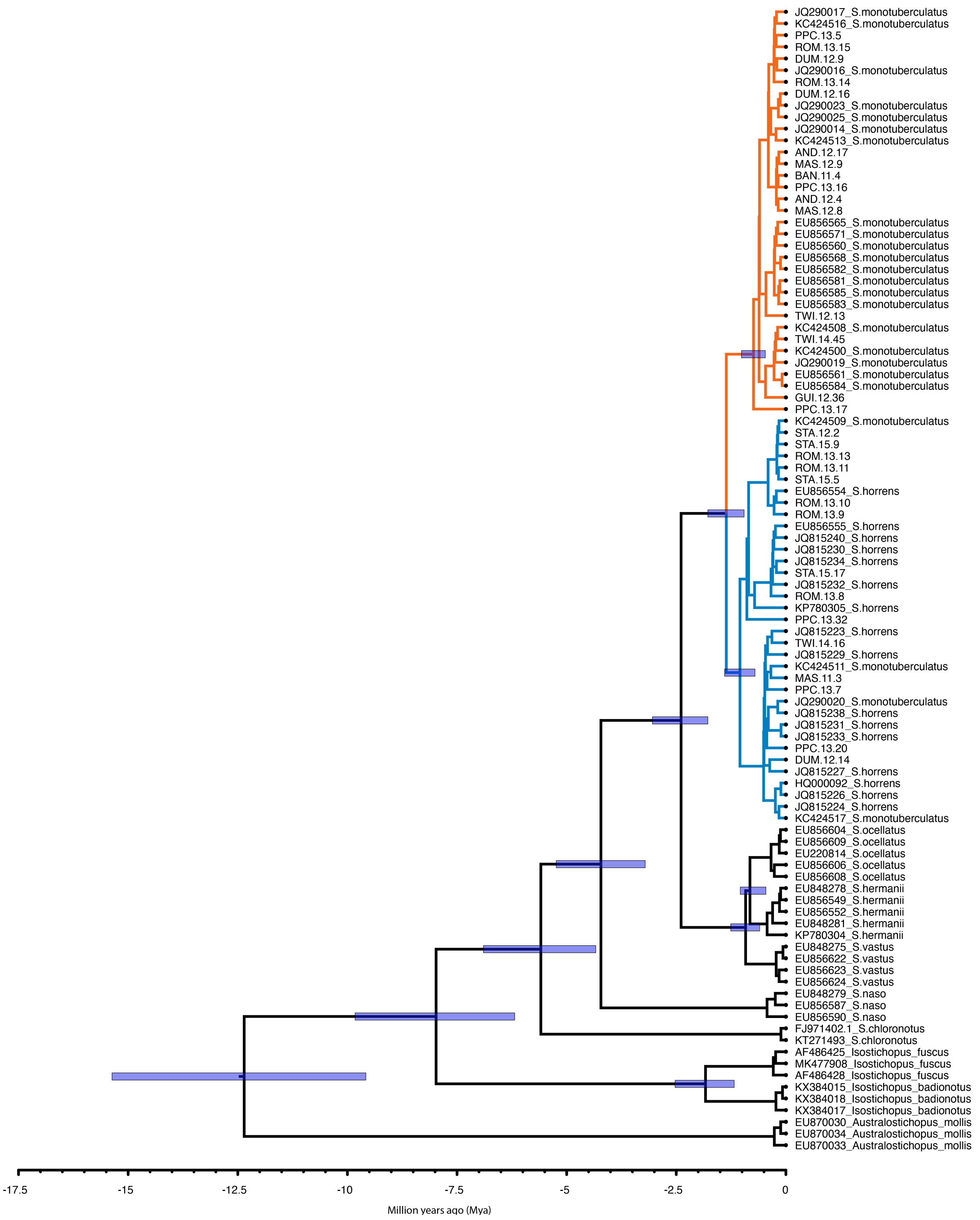

Supplementary Figure S3. Chronogram of *Stichopus* cf. *horrens, S. horrens,* and *S. monotuberculatus* resulting from analysis of the mitochondrial cytochrome oxidase I region in BEAST, displaying mean divergence time and 95% confidence interval (blue bar). Timescale is based on a substitution rate of 3.39% per million years.

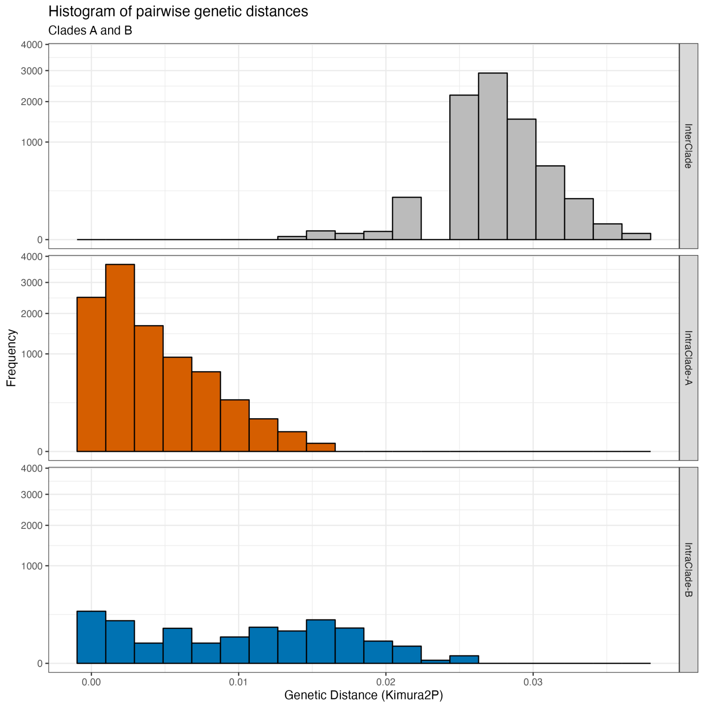

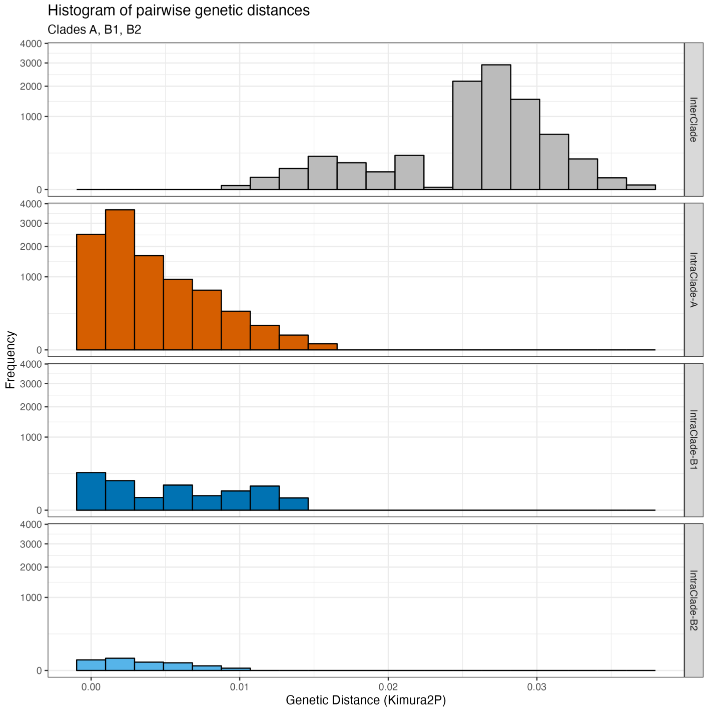

Supplementary Figure S4. Histogram of inter-clade and intra-clade pairwise distances among COI sequences of *Stichopus* cf. *horrens* lineages: Clades A and B (top); Clades A, B1, and B2 (bottom).

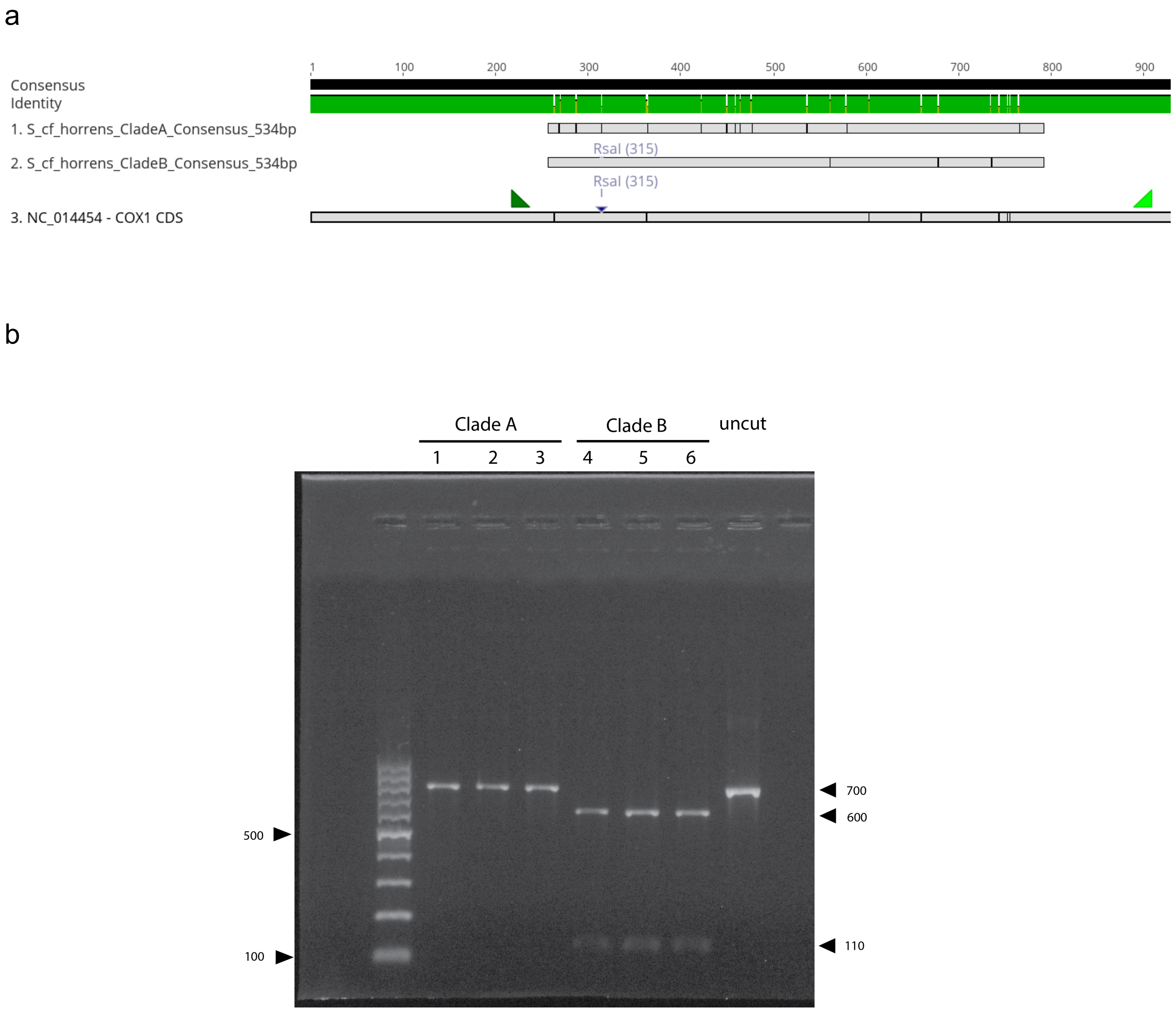

Supplementary Figure S5. (a) Schematic of the *Stichopus* cf. *horrens* mitochondrial cytochrome oxidase I region showing the COIef and COIer primer binding sites (triangles) and location of recognition sites for endonuclease *Rsa*I at position 315. Image generated using Geneious v2023.1. The first two sequences are consensus sequences for *S.* cf. *horrens* Clade A and Clade B, respectively. The third sequence is the CoI gene region from positions 1-900 extracted from the full mitochondrial genome sequence of GenBank accession NC014454 (*Stichopus horrens*). The restriction site for *Rsa*I is present in *S.* cf. *horrens* Clade B but not Clade A. (b) Image of agarose gel of the PCR-RFLP assay showing profiles diagnostic for *Stichopus* cf. *horrens* Clade A (lanes 1 – 3, ~700 bp; uncut due to absence of *Rsa*I site) and Clade B (lanes 4-6, ~600 bp and 110 bp, resulting from cleavage at COI gene position 315 or amplicon position 110).

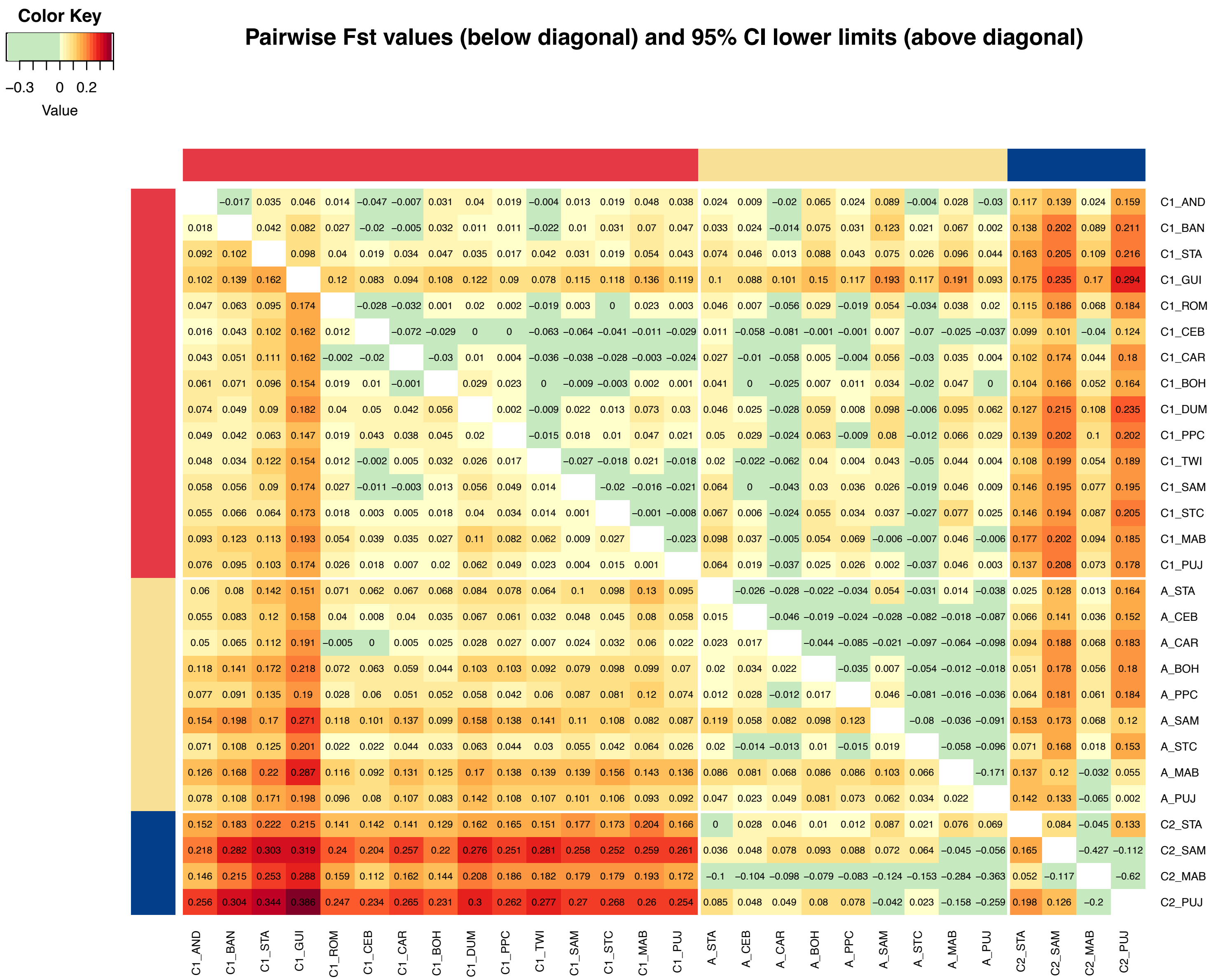

	C1_AND	C1_BAN	C1_STA	C1_GUI	C1_ROM	C1_CEB	C1_CAR	C1_BOH	C1_DUM	C1_PPC	C1_TWI	C1_SAM	C1_STC	C1_MAB	C1_PUJ	A_STA	A_CEB	A_CAR	A_BOH	A_PPC	A_SAM	A_STC	A_MAB	A_PUJ	C2_STA	C2_SAM	C2_MAB	C2_PUJ
C1_AND		−0.017	0.035	0.046	0.014	−0.047	−0.007	0.031	0.04	0.019	−0.004	0.013	0.019	0.048	0.038	0.024	0.009	−0.02	0.065	0.024	0.089	−0.004	0.028	−0.03	0.117	0.139	0.024	0.159
C1_BAN	0.018		0.042	0.082	0.027	−0.02	−0.005	0.032	0.011	0.011	−0.022	0.01	0.031	0.07	0.047	0.033	0.024	−0.014	0.075	0.031	0.123	0.021	0.067	0.002	0.138	0.202	0.089	0.211
C1_STA	0.092	0.102		0.098	0.04	0.019	0.034	0.047	0.035	0.017	0.042	0.031	0.019	0.054	0.043	0.074	0.046	0.013	0.088	0.043	0.075	0.026	0.096	0.044	0.163	0.205	0.109	0.216
C1_GUI	0.102	0.139	0.162		0.12	0.083	0.094	0.108	0.122	0.09	0.078	0.115	0.118	0.136	0.119	0.1	0.088	0.101	0.15	0.117	0.193	0.117	0.191	0.093	0.175	0.235	0.17	0.294
C1_ROM	0.047	0.063	0.095	0.174		−0.028	−0.032	0.001	0.02	0.002	−0.019	0.003	0	0.023	0.003	0.046	0.007	−0.056	0.029	−0.019	0.054	−0.034	0.038	0.02	0.115	0.186	0.068	0.184
C1_CEB	0.016	0.043	0.102	0.162	0.012		−0.072	−0.029	0	0	−0.063	−0.064	−0.041	−0.011	−0.029	0.011	−0.058	−0.081	−0.001	−0.001	0.007	−0.07	−0.025	−0.037	0.099	0.101	−0.04	0.124
C1_CAR	0.043	0.051	0.111	0.162	−0.002	−0.02		−0.03	0.01	0.004	−0.036	−0.038	−0.028	−0.003	−0.024	0.027	−0.01	−0.058	0.005	−0.004	0.056	−0.03	0.035	0.004	0.102	0.174	0.044	0.18
C1_BOH	0.061	0.071	0.096	0.154	0.019	0.01	−0.001		0.029	0.023	0	−0.009	−0.003	0.002	0.001	0.041	0	−0.025	0.007	0.011	0.034	−0.02	0.047	0	0.104	0.166	0.052	0.164
C1_DUM	0.074	0.049	0.09	0.182	0.04	0.05	0.042	0.056		0.002	−0.009	0.022	0.013	0.073	0.03	0.046	0.025	−0.028	0.059	0.008	0.098	−0.006	0.095	0.062	0.127	0.215	0.108	0.235
C1_PPC	0.049	0.042	0.063	0.147	0.019	0.043	0.038	0.045	0.02		−0.015	0.018	0.01	0.047	0.021	0.05	0.029	−0.024	0.063	−0.009	0.08	−0.012	0.066	0.029	0.139	0.202	0.1	0.202
C1_TWI	0.048	0.034	0.122	0.154	0.012	−0.002	0.005	0.032	0.026	0.017		−0.027	−0.018	0.021	−0.018	0.02	−0.022	−0.062	0.04	0.004	0.043	−0.05	0.044	0.004	0.108	0.199	0.054	0.189
C1_SAM	0.058	0.056	0.09	0.174	0.027	−0.011	−0.003	0.013	0.056	0.049	0.014		−0.02	−0.016	−0.021	0.064	0	−0.043	0.03	0.036	0.026	−0.019	0.046	0.009	0.146	0.195	0.077	0.195
C1_STC	0.055	0.066	0.064	0.173	0.018	0.003	0.005	0.018	0.04	0.034	0.014	0.001		−0.001	−0.008	0.067	0.006	−0.024	0.055	0.034	0.037	−0.027	0.077	0.025	0.146	0.194	0.087	0.205
C1_MAB	0.093	0.123	0.113	0.193	0.054	0.039	0.035	0.027	0.11	0.082	0.062	0.009	0.027		−0.023	0.098	0.037	−0.005	0.054	0.069	−0.006	−0.007	0.046	−0.006	0.177	0.202	0.094	0.185
C1_PUJ	0.076	0.095	0.103	0.174	0.026	0.018	0.007	0.02	0.062	0.049	0.023	0.004	0.015	0.001		0.064	0.019	−0.037	0.025	0.026	0.002	−0.037	0.046	0.003	0.137	0.208	0.073	0.178
A_STA	0.06	0.08	0.142	0.151	0.071	0.062	0.067	0.068	0.084	0.078	0.064	0.1	0.098	0.13	0.095		−0.026	−0.028	−0.022	−0.034	0.054	−0.031	0.014	−0.038	0.025	0.128	0.013	0.164
A_CEB	0.055	0.083	0.12	0.158	0.04	0.008	0.04	0.035	0.067	0.061	0.032	0.048	0.045	0.08	0.058	0.015		−0.046	−0.019	−0.024	−0.028	−0.082	−0.018	−0.087	0.066	0.141	0.036	0.152
A_CAR	0.05	0.065	0.112	0.191	−0.005	0	0.005	0.025	0.028	0.027	0.007	0.024	0.032	0.06	0.022	0.023	0.017		−0.044	−0.085	−0.021	−0.097	−0.064	−0.098	0.094	0.188	0.068	0.183
A_BOH	0.118	0.141	0.172	0.218	0.072	0.063	0.059	0.044	0.103	0.103	0.092	0.079	0.098	0.099	0.07	0.02	0.034	0.022		−0.035	0.007	−0.054	−0.012	−0.018	0.051	0.178	0.056	0.18
A_PPC	0.077	0.091	0.135	0.19	0.028	0.06	0.051	0.052	0.058	0.042	0.06	0.087	0.081	0.12	0.074	0.012	0.028	−0.012	0.017		0.046	−0.081	−0.016	−0.036	0.064	0.181	0.061	0.184
A_SAM	0.154	0.198	0.17	0.271	0.118	0.101	0.137	0.099	0.158	0.138	0.141	0.11	0.108	0.082	0.087	0.119	0.058	0.082	0.098	0.123		−0.08	−0.036	−0.091	0.153	0.173	0.068	0.12
A_STC	0.071	0.108	0.125	0.201	0.022	0.022	0.044	0.033	0.063	0.044	0.03	0.055	0.042	0.064	0.026	0.02	−0.014	−0.013	0.01	−0.015	0.019		−0.058	−0.096	0.071	0.168	0.018	0.153
A_MAB	0.126	0.168	0.22	0.287	0.116	0.092	0.131	0.125	0.17	0.138	0.139	0.139	0.156	0.143	0.136	0.086	0.081	0.068	0.086	0.086	0.103	0.066		−0.171	0.137	0.12	−0.032	0.055
A_PUJ	0.078	0.108	0.171	0.198	0.096	0.08	0.107	0.083	0.142	0.108	0.107	0.101	0.106	0.093	0.092	0.047	0.023	0.049	0.081	0.073	0.062	0.034	0.022		0.142	0.133	−0.065	0.002
C2_STA	0.152	0.183	0.222	0.215	0.141	0.142	0.141	0.129	0.162	0.165	0.151	0.177	0.173	0.204	0.166	0	0.028	0.046	0.01	0.012	0.087	0.021	0.076	0.069		0.084	−0.045	0.133
C2_SAM	0.218	0.282	0.303	0.319	0.24	0.204	0.257	0.22	0.276	0.251	0.281	0.258	0.252	0.259	0.261	0.036	0.048	0.078	0.093	0.088	0.072	0.064	−0.045	−0.056	0.165		−0.427	−0.112
C2_MAB	0.146	0.215	0.253	0.288	0.159	0.112	0.162	0.144	0.208	0.186	0.182	0.179	0.179	0.193	0.172	−0.1	−0.104	−0.098	−0.079	−0.083	−0.124	−0.153	−0.284	−0.363	0.052	−0.117		−0.62
C2_PUJ	0.256	0.304	0.344	0.386	0.247	0.234	0.265	0.231	0.3	0.262	0.277	0.27	0.268	0.26	0.254	0.085	0.048	0.049	0.08	0.078	−0.042	0.023	−0.158	−0.259	0.198	0.126	−0.2	

Supplementary Figure S6. Matrix of pairwise F_{ST} values (below diagonal) and lower limit of 95% CI (above diagonal) for *Stichopus* cf. *horrens* grouped according to genotype cluster. Row and column labels refer to genotype cluster (C1 = Cluster 1, C2 = Cluster 2, A = Admixed) and collection site (Site Code as in Table 1). Color bars beside row and column labels indicate cluster (Red = Cluster 1, Yellow = Admixed, Blue = Cluster 2). Cells are colored according to value (color key). F_{ST} and lower limit of 95% CI values < 0 are colored in green and values > 0 are colored from yellow to red. 95% CI values < 0 (green cells), indicate nonsignificant genetic variation ($F_{ST} = 0$), while values > 0 indicate significant genetic variation ($F_{ST} > 0$).
