# Supplementary material for: Pseudocryptic diversity and species boundaries in the sea cucumber Stichopus cf. horrens (Echinodermata: Stichopodidae) revealed by mitochondrial and microsatellite markers
Source: Sci Rep. 2024 Feb 28;14:4886. doi: 10.1038/s41598-024-54987-w (PMC10901784; doi:10.1038/s41598-024-54987-w)
Supplement: Supplementary file 3 — Supplementary Information 3. [file 41598_2024_54987_MOESM3_ESM.pdf]

Supplementary Table S2. Haplotype frequency distribution of *Stichopus horrens* and *Stichopus monotuberculatus* GenBank accessions and *Stichopus cf. horrens sequences* (this study), summarized by sample location

Haplotype	Label	Clade	SubClade	Indian Ocean	Strait of Malacca	South China Sea	Philippines, South China Sea	Philippine Sea	Sulu Sea	Bohol Sea	Sibuyan Sea	Celebes Sea	Davao Gulf	Northeast Australia	South Pacific	ND	Total
4	BAN_11_11	A	A		3	4	3	23	4	6	5	4	16		2		70
6	DUM_12_4	A	A			13	2	1	8	7	3						34
1	AND_12_2	A	A			1	20					2					23
5	DUM_12_1	A	A		1	4				3	3		5				16
60	EU856582_Smonotuberculatus	A	A											3	8		11
11	GUI_12_36	A	A			2		7									9
53	KY986418_Shorrens	A	A		4	2											6
3	BAN_11_4	A	A		1		3										4
62	EU856565_Smonotuberculatus	A	A											3			3
66	EU856585_Smonotuberculatus	A	A											3			3
70	EU856561_Smonotuberculatus	A	A											3			3
34	TWI_12_2	A	A									2					2
65	EU856581_Smonotuberculatus	A	A											2			2
68	KC424500_Smonotuberculatus	A	A			2											2
69	EU856584_Smonotuberculatus	A	A											2			2
2	AND_12_4	A	A				1										1
7	DUM_12_9	A	A							1							1
10	DUM_12_16	A	A							1							1
13	MAS_12_8	A	A				1										1
14	MAS_12_9	A	A				1										1
15	PPC_13_5	A	A						1								1
17	PPC_13_16	A	A						1								1
18	PPC_13_17	A	A						1								1
27	ROM_13_14	A	A								1						1
28	ROM_13_15	A	A								1						1
37	TWI_14_45	A	A									1					1
55	JQ290017_Smonotuberculatus	A	A			1											1
56	JQ290025_Smonotuberculatus	A	A			1											1
57	JQ290016_Smonotuberculatus	A	A			1											1
58	JQ290014_Smonotuberculatus	A	A			1											1
59	EU856568_Smonotuberculatus	A	A											1			1
61	EU856560_Smonotuberculatus	A	A											1			1
63	EU856571_Smonotuberculatus	A	A											1			1
64	EU856583_Smonotuberculatus	A	A											1			1
67	JQ290019_Smonotuberculatus	A	A			1											1
21	PPC_13_33	B	B1			4		11	1		2	6	3				27
31	STA_15_3	B	B1			1		3									4
33	STA_15_17	B	B1			2		2									4
20	PPC_13_32	B	B1						1			2					3
23	ROM_13_9	B	B1			1					1	1					3
24	ROM_13_10	B	B1					1			1	1					3
22	ROM_13_8	B	B1					1			1						2
25	ROM_13_11	B	B1								1						1
26	ROM_13_13	B	B1								1						1
29	STA_12_2	B	B1					1									1
32	STA_15_9	B	B1					1									1
39	EU856555_Shorrens	B	B1												1		1
40	JQ815240_Shorrens	B	B1			1											1
41	JQ815234_Shorrens	B	B1			1											1
42	JQ815230_Shorrens	B	B1			1											1
43	JQ815232_Shorrens	B	B1			1											1
44	EU856554_Shorrens	B	B1											1			1
8	DUM_12_10	B	B2			4		1		1	1	4				1	12
30	STA_14_4	B	B2			4		1									5
35	TWI_12_9	B	B2			3						1					4
9	DUM_12_14	B	B2							1							1
12	MAS_11_3	B	B2				1										1
16	PPC_13_7	B	B2						1								1
19	PPC_13_20	B	B2						1								1
36	TWI_14_16_Sth	B	B2									1					1
38	KP780305_Shorrens	B	B2	1													1
45	HQ000092_Shorrens	B	B2			1											1
46	JQ815229_Shorrens	B	B2			1											1
47	JQ815231_Shorrens	B	B2			1											1
48	JQ815233_Shorrens	B	B2			1											1
49	JQ815238_Shorrens	B	B2			1											1
50	JQ815227_Shorrens	B	B2			1											1
51	JQ815226_Shorrens	B	B2			1											1
52	JQ815224_Shorrens	B	B2			1											1
54	JQ290020_Smonotuberculatus	B	B2			1											1
				1	9	65	32	53	19	20	21	25	24	21	11	1	302

Supplementary Table S3. Cross-tabulation of STRUCTURE and NewHybrids assignment of 396 *Stichopus* cf. *horrens* into genotype clusters and hybrid categories, respectively, based on six microsatellite loci. Highlighted cells indicate concordant assignments between STRUCTURE and NewHybrids.

	**NewHybrids Category**			
**STRUCTURE Cluster**	**Parental 1**	**Parental 2**	**F2**	**Unassigned**
Cluster 1	282	None	4	1
Cluster 2	None	19	22	2
Admixed	15	None	51	None

Supplementary Table S4. Per-locus F_{ST} values and 95% confidence intervals (95% CI) for *Stichopus* cf. *horrens* (n = 396 individuals) when grouped by microsatellite genotype cluster: Cluster 1 (n = 273 individuals), Cluster 2 (n = 43 individuals, Admixed (n = 66 individuals).

**Locus**	F_{ST}	F_{ST} **95% CI**	G'_{ST}	G'_{ST} **95% CI**
Sm001	0.0410	0.0212 – 0.0655	0.1211	0.0579 – 0.2003
Sm007	0.0152	0.0045 – 0.0289	0.1818	0.0757 – 0.3041
Sm010	0.2878	0.2449 – 0.3338	0.6846	0.6378 – 0.7354
Sm011	0.0154	0.0074 – 0.0247	0.2062	0.1053 – 0.3120
Sm012	0.0043	-0.0012 – 0.0112	0.0598	- 0.0158 – 0.1465
Sm013	0.1243	0.0931 – 0.1603	0.4762	0.3930 – 0.5679

Supplementary Table S5. Genetic diversity estimates for three genotype clusters of *Stichopus* cf. *horrens* at six microsatellite loci. Number of individuals analyzed (N), number of alleles (A), number of private alleles (A_P), number of shared alleles (A_S), allelic richness (A_R), observed heterozygosity (H_O), expected heterozygosity (H_E), inbreeding coefficient (F_{IS}), and Hardy-Weinberg equilibrium p-value (HWE *P*) are indicated. Significant *P* values ($P < 0.05$) following a table-wide Bonferroni correction ($P < 0.0027$) are indicated in bold.

	Sm001	***Sm007***	***Sm010***	***Sm011***	***Sm012***	***Sm013***
**Cluster 1**						
N	287	287	287	287	287	287
A	9	18	17	34	26	19
A_P	1	3	5	10	5	6
A_S	8	15	12	24	21	13
A_R	6.68	14.54	11.51	20.59	19.81	12.33
H_O	0.505	0.676	0.425	0.77	0.951	0.627
H_E	0.637	0.866	0.494	0.908	0.925	0.785
F_{IS}	0.208	0.221	0.142	0.154	-0.027	0.203
HWE *P*	0.024	**0.000**	**0.000**	**0.000**	0.915	**0.000**
**Cluster 2**						
N	43	43	43	43	43	43
A	8	17	11	23	17	12
A_P	1	1	2	2	1	3
A_S	7	16	9	21	16	9
A_R	7.97	17	10.48	22.65	16.91	11.14
H_O	0.763	0.649	0.254	0.823	0.94	0.212
H_E	0.79	0.892	0.67	0.935	0.909	0.464
F_{IS}	0.043	0.28	0.626	0.129	-0.027	0.548
HWE *P*	0.431	**0.000**	**0.000**	**0.000**	0.493	**0.000**
**Admixed**						
N	66	66	66	66	66	66
A	12	18	13	32	22	17
A_P	3	1	2	5	2	1
A_S	9	17	11	27	20	16
A_R	10.68	17.3	11.07	27.37	20.2	15.44
H_O	0.614	0.447	0.562	0.82	0.841	0.593
H_E	0.794	0.884	0.751	0.937	0.924	0.817
F_{IS}	0.232	0.499	0.258	0.13	0.096	0.279
HWE *P*	**0.000**	**0.000**	**0.000**	0.007	**0.001**	**0.000**
